# Supplementary material for: Unveiling patterns in human dominated landscapes through mapping the mass of US built structures
Source: Nat Commun. 2023 Dec 4;14:8014. doi: 10.1038/s41467-023-43755-5 (PMC10695923; doi:10.1038/s41467-023-43755-5)
Supplement: Supplementary file 1 — Supplementary Information [file 41467_2023_43755_MOESM1_ESM.pdf]

Supplementary Information for

Unveiling patterns in human dominated landscapes through mapping the mass of US built structures

David Frantz, Franz Schug, Dominik Wiedenhofer, André Baumgart, Doris Virág, Sam Cooper, Camila Gómez-Medina, Fabian Lehmann, Thomas Udelhoven, Sebastian van der Linden, Patrick Hostert, Helmut Haberl

Correspondence to: david.frantz@uni-trier.de

This PDF file includes:

Supplementary Methods 1-5

Supplementary Notes 1

Supplementary Discussion 1-5

Supplementary Figures 1 to 10

Supplementary Tables 1 to 20

Supplementary References

Table of Contents

Supplementary Methods 1. Processing of Earth observation data 2
Supplementary Methods 2. Conversion to mass factors per volume 3
Supplementary Methods 3. Uncertainty of material factors 4
Supplementary Methods 4. Relationship between building and mobility infrastructure density 5
Supplementary Methods 5. Multivariate regression 5
Supplementary Notes 1. Definition: urban / rural 7
Supplementary Discussion 1. Quality of building footprints 8
Supplementary Discussion 2. Quality of building height prediction 8
Supplementary Discussion 3. Quality of building type prediction 9
Supplementary Discussion 4. Geographic origin of material factors for buildings 10
Supplementary Discussion 5. Remaining impervious areas 10
Supplementary Figure 1. Conceptual overview 12
Supplementary Figure 2. Area correction factor 13
Supplementary Figure 3. Building height hold-out validation 14
Supplementary Figure 4. Building climate zones 15
Supplementary Figure 5. Building volume definition and material factor unit conversion 16
Supplementary Figure 6. Road definition 17
Supplementary Figure 7. Road climate zones 18
Supplementary Figure 8. Railway definition 19
Supplementary Figure 9. Intercomparison of total mapped material stocks 20
Supplementary Figure 10. Intercomparison of mapped stocks in buildings and roads 21
Supplementary Table 1. Predictive variables building height 22
Supplementary Table 2. Predictive variables building types 23
Supplementary Table 3. Building height cross-validation and data sources 24
Supplementary Table 4. Building types 25
Supplementary Table 5. Intermediate building types 25
Supplementary Table 6. Building type training sites 26
Supplementary Table 7. Quality of building type prediction 26
Supplementary Table 8. Building climate zones 27
Supplementary Table 9. Mass factors for buildings 28
Supplementary Table 10. Road types and buffer widths 29
Supplementary Table 11. Mass factors for roads 31
Supplementary Table 12. Road climate zones 32
Supplementary Table 13. Railway types and buffer widths 32
Supplementary Table 14. Mass factors for railways 33
Supplementary Table 15. Mass factors for airport and parking infrastructure 33
Supplementary Table 16. Composition of remaining imperviousness areas 34
Supplementary Table 17. Socio-economic variables 35
Supplementary Table 18. Multivariate linear regression results for buildings 36
Supplementary Table 19. Multivariate linear regression results for mobility infrastructure 36
Supplementary Table 20. Collection of supplementary items describing uncertainty 37
Supplementary References 38

Supplementary Methods 1. Processing of Earth observation data

The satellite data processing follows the description in [1], but is shortly summarized here for completeness.

The Sentinel-1 constellation provides all-weather, day-and-night C-band Synthetic Aperture Radar (SAR) backscatter observations at 10 m spatial gridding [2], providing a same-orbit revisit frequency of 6 days. In this study we used ground range detected (GRD) and high-resolution (HR) Interferometric Wide (IW) swath scenes for the year 2017. This dataset covers a 250 km swath and contains backscatter intensities at 10 m pixel spacing in vertical-vertical (VV) and vertical-horizontal (VH) polarization. Where possible, data from both orbit directions were combined to enlarge the observation space for maximization of information content and reduction of areas in radar shadows [3]. The pre-processing to backscatter values (sigma-naught in dB) was orchestrated with the SAR Geophysical Retrieval Toolbox (SGRT), and the Sentinel Application Platform (SNAP) and the 3-arc seconds SRTM terrain model [4] were used for radiometric calibration and Range Doppler geometric terrain correction. Border noise effects were eliminated with the bidirectional all-samples approach introduced by [5]. The Sentinel-1 data for 2017 were provided in-kind by Technical University Vienna through the Earth Observation Data Centre (EODC, Vienna, Austria).

The Sentinel-2 constellation provides multi-spectral optical observations in 13 wavelengths at up to 10 m spatial resolution, providing a nadir revisit frequency of 5 days [6]. Due to the wide swaths of 290 km, lateral image overlaps can be considerable and partially increase revisit frequency to 2-3 days. We used all Level 1C products acquired in 2018 with cloud coverage < 50%. The Sentinel-2 constellation was fully ramped up on 17 February 2018, thus providing full data density from this date. We generated Level 2 Analysis Ready Data (ARD) through the Framework for Operational Radiometric Correction for Environmental monitoring (FORCE) [7]. Clouds and cloud shadows were identified using a modified version of the Fmask algorithm [8–12]. All images were radiometrically standardized [12] using radiative transfer modelling-based atmospheric correction [13], topographic correction [14], adjacency effect correction, and nadir BRDF-adjustment [15,16]. The 20 m bands were enhanced to 10 m resolution using a data fusion approach [17], and only the 10 land application bands covering VIS, NIR and SWIR domains were retained. Both Sentinel-1 backscatter and Sentinel-2 reflectance were aligned with the EQUI7 grid [18].

All EO data were accessed from the data archive of the Earth Observation Data Centre (EODC, https://eodc.eu/), which provides a petabyte-scale storage that contains amongst other satellite data the complete worldwide Sentinel-1 IW and Sentinel-2 L1C data record. The EODC infrastructure is coupled with the supercomputing resources of the Vienna Scientific Cluster (VSC), on which the bulk preprocessing was performed.

We derived Spectral Temporal Metrics (STM) [19–21] from the preprocessed data to retain temporal information while reducing data volume considerably. STMs are statistical aggregations of all available high-quality observations within a specified time period (one year in this case), and as such provide rich information on spectral-temporal variability and data distribution [7]. Due to their spatial completeness and fairly high robustness against different observation densities, they are suitable features for machine learning applications, and have proven to be effective for a large variety of land cover mapping or quantitative variable estimation [e.g., 22,23]. For the optical time series, only clear-sky, non-cloud, non-cloud shadow, non-snow observations were considered, which were further passed through an outlier detection routine. For the radar time series, each observation was considered. The STMs were generated separately for each band of the SAR (two bands) and optical time series (10 bands), as well as for several spectral indices. To capture the spatial characteristics originating from shadows cast off from nearby buildings as well as settlement structure, texture metrics are an established means to capture spatial context in image processing [24]. We computed texture metrics on top of the STMs with a circular structuring element with a 50 m radius – denoted as spatial STM (SSTM) [1]. A list of employed STMs and SSTMs is given Supplementary Table 1 and Supplementary Table 2 for building height and building type mapping, respectively.

Supplementary Methods 2. Conversion to mass factors per volume

In the literature, material stocks in buildings are usually reported as either absolute mass per building unit, or as materials per square meter of useful floor area. Because this study derives the *above-ground building volumes* as three-dimensional LOD1 blocks (see also volume definition in [25]) from Earth Observation data, a re-estimation and transformation of published information was required to match the definition depicted in Supplementary Figure 5.

The following equations summarize our conversion approach; each given equation is individually applied to each building type with building type-specific values. In-depth details can be found in [26]. When building volumes were not reported in studies, they were calculated based on available data by dividing the gross floor area gfa [m²] by the number of floors nf to obtain the gross footprint gf [m²]:

$$\mathrm{gf} = \mathrm{gfa}/\mathrm{nf} \quad (1)$$

If only the usable floor area ua [m²] was reported, a factor for the assumed share of the useable area in the gross floor area sua [%] provided by [26] was used:

$$\mathrm{gf} = \mathrm{ua} \cdot (\mathrm{sua}/100) \quad (2)$$

Subsequently, the above ground building height agbh [m] was calculated by multiplying the number of floors nf by the floor height fh [m]. If floor heights were not listed, values provided by [26] or in similar U.S. case studies were used.

$$\text{agbh} = \text{nf} \cdot \text{fh} \tag{3}$$

The above-ground building volume (excluding the roof) agbv [m³] was then calculated by multiplying the gross footprint [m²] by the above-ground building height [m]:

$$\text{agbv} = \text{gf} \cdot \text{agbh} \tag{4}$$

To account for the usually unreported roof volume of some building types such as low-rise single- and multi-family houses, the gross footprint was multiplied by the roof volume factor provided by [27]. The sum of the above ground volume (excluding the roof) and the roof volume corresponds with the building volume that is mapped via Earth Observation data. The material quantities reported in case studies were then divided by the above ground volume, producing the final mass factors. Supplementary Table 9 shows the developed mass factors per building type and climate zone in four broad material categories. These four material categories were further differentiated into 15 specific materials, which we published as supplementary data to this article [28], i.e. (i) metals: iron and steel, copper, aluminum, and other metals; (ii) non-metallic minerals: concrete, bricks, glass, aggregate, and other minerals; (iii) biomass-based materials: timber and other biomass-based materials such as boards; (iv) petrochemical-based materials: bitumen and other petrochemical-based materials; and (v) other materials: insulation materials and all other materials.

Supplementary Methods 3. Uncertainty of material factors

Average material factors (Supplementary Table 9, Supplementary Table 11, Supplementary Table 14, Supplementary Table 15) were used for the final material stock calculation. For uncertainty assessment purposes, these average factors were supplemented with low and high estimate material factors, representing the interquartile range (25th and 75th percentile) of all data points per stock type and material. Low and high estimate material intensity factors were used for all stock types except for those infrastructure types where only one data source was used, namely motorways, road bridges and tunnels, all rail-based infrastructure except for railway, and airport runways.

For local and rural roads, low and high estimates were likewise prepared. In a first step, variation in local road material intensity factors were used for both local roads and in the weighting of paved rural roads. In a second step, both the assumed share of unpaved roads in local roads as well as the assumed share of dirt roads in unpaved roads was increased or reduced by 15% for low and high estimates respectively. Total

uncertainty of the complete stock, and that of buildings and mobility infrastructures, were computed using 10,000 random combinations of low, average, and high material factors, eventually computing the standard deviation of the 10,000 sums. This resulted in an estimated uncertainty of 5.8 Gt, 4.7 Gt, and 3.5 Gt for the total, building, and mobility infrastructure stock, respectively.

Supplementary Methods 4. Relationship between building and mobility infrastructure density

To analyze the relationship between buildings and mobility infrastructure stocks per area on the county level, a linear Ordinary Least Squares (OLS) regression model was employed using R 4.2.0 and the stats package [29]. Both variables were log-transformed to shift their frequency distributions towards normality. A highly significant relationship (p < 2e-16, n = 3,108) was found with $R^2 = 0.88$. Note however, that the high significance is substantially affected by the large sample size.

Supplementary Methods 5. Multivariate regression

To link material consumption of buildings and mobility infrastructure to possibly explanatory variables on the county-level (Supplementary Table 17), multivariate regression analyses were carried out using R 4.2.0 and the stats and spdep packages [29,30].

Both target variables (i.e., *material intensity of buildings, and material intensity of mobility infrasructures* in t cap^{-1}*)* were log-transformed to shift their frequency distributions towards normality. Population density and per-capita GDP were log-transformed, too. All dependent and independent variables were z-score normalized scaled to allow a comparison of the final regression coefficients of the models.

A Moran test indicated strong spatial autocorrelation effects (p=0.001) in the two target variables.

The statistical analysis was accomplished in two steps. First, for both target variables, a variable selection was performed using stepwise multivariate regression using the Bayesian Information Criterion (BIC) as selection criterion. To control the spatial autocorrelation problem in this stage, a random subsample of 500 observations from our original dataset (n = 3108) were drawn to reduce the likelihood that results were influenced by spatially clustered points. In a second step, the identified relevant predictor variables were used to fit for each target variable a spatial autoregressive model to the complete dataset using the spdep::sacsarlm R-function, to account for the full spatial dependency in the dependent variables (Bivant et al., 2013). This model has the form

$$y = \rho \boldsymbol{W} y + \boldsymbol{X\beta} + u, \quad (5)$$

$$u = \lambda \boldsymbol{W} u + \varepsilon, \quad (6)$$

where:

y = dependent variable

$\boldsymbol{X}$ = matrix of independent variables

$\boldsymbol{\beta}$ = vector of coefficients for the independent variables

u = (autocorrelated) error term

$\boldsymbol{W}$ = spatial weights matrix, which defines the neighborhood structure based on regions with contiguous boundaries

λ = spatial autoregressive parameter for the error term

ε = residual term

A sacsarlm model can capture complex spatial relationships by combining a Spatial Error Model (SEM) that assumes a moving average process of the spatially lagged error term and a Spatial Autoregressive Model (SAR) that can handle a spatial autoregressive process by including lagged effects of the dependent variable. In the equation ρ captures the spatial autocorrelation in the dependent variable (y), and λ captures the spatial autocorrelation in the error term (u).

The model outcomes (Supplementary Table 18, Supplementary Table 19) suggest that all of the included predictors have significant statistical associations with material intensity, which is also triggered by the large sample size (>3000 observations). Therefore, it is also mandatory to consider the effect sizes. The outcome of the Moran test applied to the model residuals indicates that both models have effectively accounted for the spatial structure in the data.

As the SAR model could not produce reliable Pseudo-R^2 values (note that the mobility infrastructure model generated an R^2 of 1.00), we further used the lrtest function from the lmtest library for carrying out a likelihood ratio test between the full SARCAR-model and a nested model without explanatory variables. A significant test result supports the assumption that the estimations of the full model provides a significantly better fit to the data and that they are not primarily based on the spatial autocorrelation structure.

Supplementary Notes 1. Definition: urban / rural

We analyze stocks, and the relative dominance of stock categories with regards to the percentage of the urban and rural population in the US counties. We follow the urban and rural classification of the US census (2010 Census Urban and Rural Classification and Urban Area Criteria https://www.census.gov/programs-surveys/geography/guidance/geo-areas/urban-rural/2010-urban-rural.html, last accessed 04.07.2023):

"For the 2010 Census, an urban area will comprise a densely settled core of census tracts and/or census blocks that meet minimum population density requirements, along with adjacent territory containing non-residential urban land uses as well as territory with low population density included to link outlying densely settled territory with the densely settled core. To qualify as an urban area, the territory identified according to criteria must encompass at least 2,500 people, at least 1,500 of which reside outside institutional group quarters. The Census Bureau identifies two types of urban areas:

- *Urbanized Areas (UAs) of 50,000 or more people;*
- *Urban Clusters (UCs) of at least 2,500 and less than 50,000 people.*

Rural encompasses all population, housing, and territory not included within an urban area."

Under the above link, the US census 2010 provides a dataset that contains the percentage of urban and rural population per county, i.e., the population living in urban or rural areas according to the definition above. The percentage of urban and rural population sums to 100%.

Supplementary Discussion 1. Quality of building footprints

Building footprint vector geometries were downloaded from https://github.com/microsoft/USBuildingFootprints (version 1.1), which were generated by Microsoft with a deep neural network trained with 5 million labeled very high-resolution Bing images (30 cm resolution). Microsoft reports a precision of 99.3% and a recall of 93.5%. An independent validation of version 1.0 of this dataset was performed by [31]; visual checks confirmed that raised issues are still apparent in version 1.1. According to Heris et al. [31], the accuracy for large building is indeed very high, but the dataset has a strong omission of small accessory buildings (< 150 m²): precision drops to 80% with decreasing area; recall drops significantly below 150 m², in some cases below 40%. This might be related to uncertainty in generating training data by manually digitizing very high-resolution data [32] or might even be implemented on purpose to avoid false positive detections of other infrastructure types that might be confused with small buildings, e.g., transportation containers or trucks. Please note that the dataset description of Microsoft is rather short. Another issue of this dataset is that connected buildings with similar height are consolidated into superblocks [31]. This does not affect our measure of building area, but our prediction of building type is partially affected by this as footprint centroid density was used as one of the predictive variables. False positives have been identified in lakes, rivers, and snow-covered areas [31], as well as in desert areas (https://github.com/microsoft/USBuildingFootprints/issues/57). We largely eliminated these false positives with a building height threshold of 2 m. False negative blocks were identified in some geographic areas, presumably due to a lack of aerial imagery [31]. These false negatives will however persist in our maps and will most likely be represented by parking and yard area, as most buildings still appear in the National Land Cover Database's imperviousness layer (see section *Remaining impervious areas*). As most unresolved issues and quality measures indicate underdetection, we rather expect that the uncertainty in the building footprint dataset results in an underestimation of building stocks.

Supplementary Discussion 2. Quality of building height prediction

The reference data were manually quality screened and homogenized. We validated the model using 1) a 70-30 data split for evaluating overall model performance (Supplementary Figure 3), and 2) a per-dataset cross-validation (Supplementary Table 3).

Most reference samples are taken from buildings shorter than 10-15 m, for which we obtained very accurate predictions close to the one-to-one line. Ground reference sample (height = 0) were also predicted with high accuracy, although some overprediction is apparent (bottom row in Supplementary Figure 3a), which however is uncritical for our final map: ground samples were incorporated into the modelling process to adapt the machine learner to non-building surfaces. All height predictions shorter than 2 m were set to 0 m

in a postprocessing step, which eliminated false positive building detections to a large degree. Saturation, i.e., underprediction, is however apparent for buildings taller than ca. 35 m (cf. Supplementary Figure 3b). This effect was also described by [1] for Germany, which the authors attributed to 1) physical limitations inherent in the employed satellite data, and 2) insufficient number of training points as high-rise buildings in Germany are rather rare. In the CONUS, high-rise buildings and skyscrapers are more commonplace, however. Our validation confirms that saturation takes effect when predicting tall buildings, which partially corroborates those physical limitations are inherent in the employed data. However, in our case, saturation takes place above ca. 35 m as compared to ca. 20 m in [1]. The better performance of the present model could be driven by both a more frequent occurrence of tall building samples and by their higher density that results in a more distinct representation in the SSTMs.

Nevertheless, saturation is still a dominant factor that precludes predicting high-rise buildings, or even skyscrapers, for which we would substantially underestimate material stocks. Thus, we additionally compiled data from the tall building database maintained by the *Council on Tall Buildings and Urban Habitat* (https://www.ctbuh.org, accessed on 03.11.2020), which contains the architectural height and coordinates of buildings taller than circa 65m. There is no information on the completeness of this database, but visual investigation suggests that the completeness increased rapidly towards the tallest buildings. As the Microsoft building footprints are subject to superblock issues, and upon visual inspection OSM completeness was found to be high for these prominent structures, we matched the coordinates of the tall building database with building geometries from OSM. We burned the database's height attribute into the EO-based building layer to better accommodate for tall buildings. For buildings not included in the tall buildings database, and larger than ca. 35 m, the EO-based underestimation of building height accordingly adds an underestimation component to our final material stocks estimation.

Supplementary Discussion 3. Quality of building type prediction

The accuracy assessment of building types is presented in Supplementary Table 7. Two major data characteristics should be discussed regarding building type mapping. First, as actual building functions and uses are nearly impossible to detect from satellite imagery, building types were mapped based on textural context indicating their potential construction type. Thus, in selected cases, e.g., a closed production site now being used for residential purposes, the validation procedure has limitations. The sampling of RES and RCMU housing was sometimes challenging in transition zones between city centers and adjacent suburban areas, especially where both types were present in the local vicinity. In general, however, the distinction of these broad building type categories worked reasonably well as RES, CI, and MLB buildings have very distinct structural characteristics in many parts of the country. Second, the building footprint data provided

an underestimation of the number of buildings in areas where many attached buildings with similar height and roof structure were merged into superblocks in the Microsoft building footprint dataset, for example in residential row development. Here, the small number of building centroids rather suggested industrial and commercial building structures instead of residential use.

Supplementary Discussion 4. Geographic origin of material factors for buildings

Preferably, studies for the United States were used to derive material factors (Supplementary Table 9). About 58% of 71 building mass factors used were sourced from building studies for the United States. For those building types where data was scarce, studies from economically comparable countries (mainly Canada and Australia) were used, where similar building standards and climate zones were assumed (ca. 32% of all mass factors). Mass factors for buildings in other countries make up the remaining 10% of all building mass factors. For the classes of high-rise buildings (RCMU-HR, RCMU-SKY), studies from East Asia and Europe were used as well, as such structures were assumed to be sufficiently homogeneous across the globe. Furthermore, the mass-per-volume factors for mobile homes and other light-weight structures are based on material quantities of post-disaster pre-manufactured housing in Turkey [33] as this case study was evaluated to be most analogous to mobile homes or structures, colloquially known as trailer parks. Different construction standards across climate zones are particularly relevant for residential buildings across the CONUS. Mass factors for this building type were therefore developed for each climate zone (Supplementary Table 8). A total of 21 mass factors from U.S. case studies and 13 mass factors from case studies outside the United States were aligned to five climate zones based on the location of each case study. For the other building types, it was assumed that construction practices and standards do not differ to an extent that would justify the use of a differentiated mass factor.

Supplementary Discussion 5. Remaining impervious areas

To safeguard our assumption that these remaining impervious surfaces are parking-related spaces or surfaces that have a similar material composition, we drew a random sample across the CONUS and manually labeled 1,000 sample points using visual interpretation of Google Earth imagery. Nearly 70% of the samples (type I in Supplementary Table 16) were indeed related to parking, yards, or similar surfaces. Five percent of the samples were impervious surfaces that were not covered by any of the other layers (type II in Supplementary Table 16), which suggests that our class catalogue is not complete and we are missing specific mass-per-area factors for these surfaces. However, given the low share of these surfaces, we believe this uncertainty to be reasonably small with regards to the entire material stocks budget. Some samples

were labeled as buildings (type III), which is presumably related to the underdetection tendency of the building footprint dataset, especially for smaller buildings. A portion of samples was found to be other mobility infrastructures (type IV), which suggests some uncertainty in the buffer procedure to convert OSM line features to polygons, e.g., when a street is wider than the design manual specification – which are defined as minimal width requirements. About 6% of the samples were natural surfaces like trees, soils, or rocks (type V), which represents an overestimation component towards our material stocks estimation.

Nevertheless, the largest share by far are parking and yard areas. Thus, we subsumed the remaining impervious area and the OSM-extracted parking spaces into the “parking and yards” class.

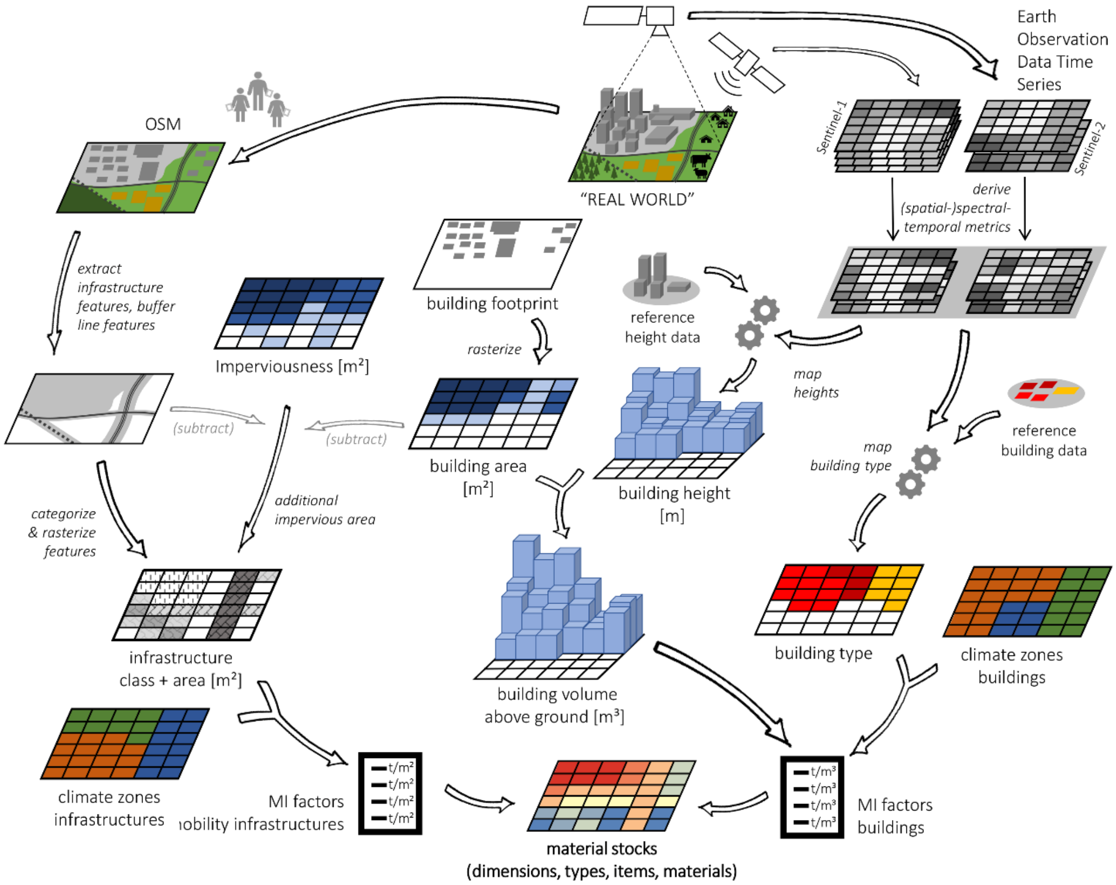

Supplementary Figure 1. Conceptual overview

Data sources used and processing workflow used to generate material stocks maps. The original version of this flowchart was printed in Haberl et al. (2021), but modified here to reflect advances in the workflow.

Haberl, H., Wiedenhofer, D., Schug, F., Frantz, D., Virág, D., Plutzar, C., Gruhler, K., Lederer, J., Schiller, G., Fishman, T., Lanau, M., Gattringer, A., Kemper, T., Liu, G., Tanikawa, H., van der Linden, S. & Hostert, P. High-Resolution Maps of Material Stocks in Buildings and Infrastructures in Austria and Germany. Environ Sci Technol 55, 3368–3379 (2021). DOI: https://doi.org/10.1021/acs.est.0c05642. Licensed under CC-BY 4.0 (https://creativecommons.org/licenses/by/4.0/)

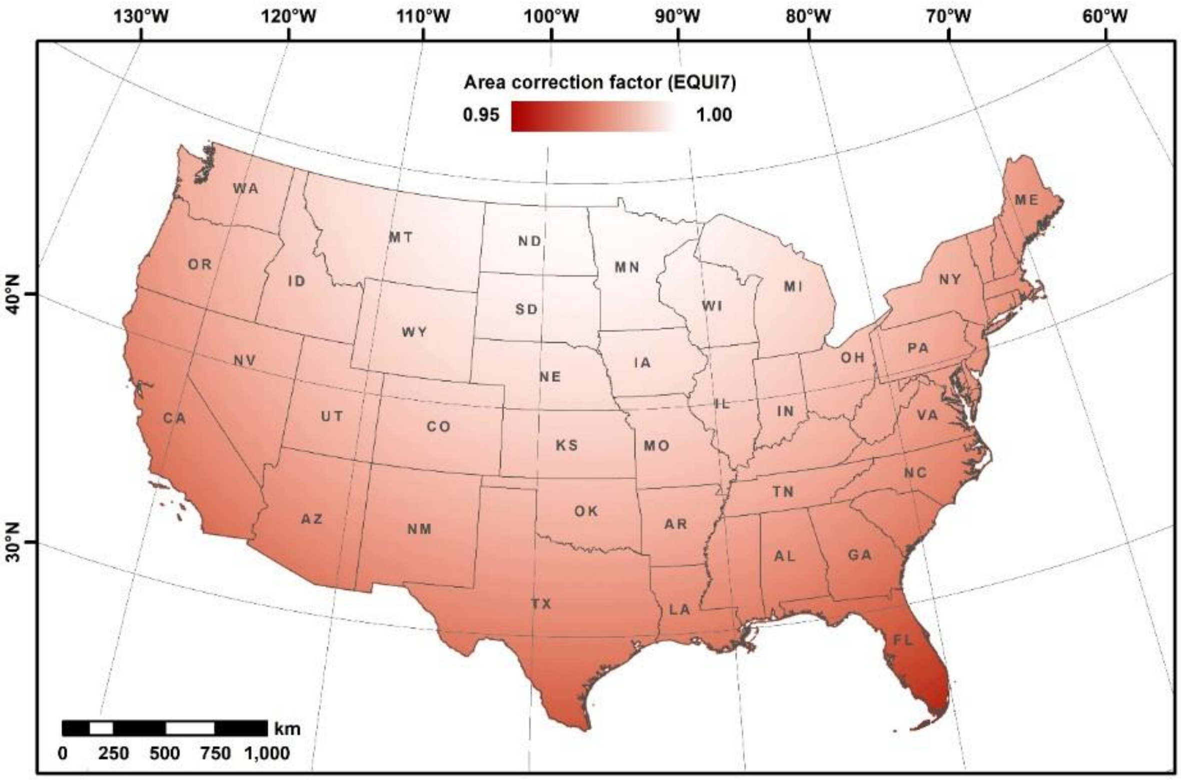

Supplementary Figure 2. Area correction factor

Area correction factor employed in this study to convert from percentage cover [%] in equidistant map projection to true area [m^2]. This correction factor varies across the CONUS, with only negligible effect in the north, but up to 4.5% correction of areal over-reporting in Florida. State boundaries were provided by the US Census Bureau.

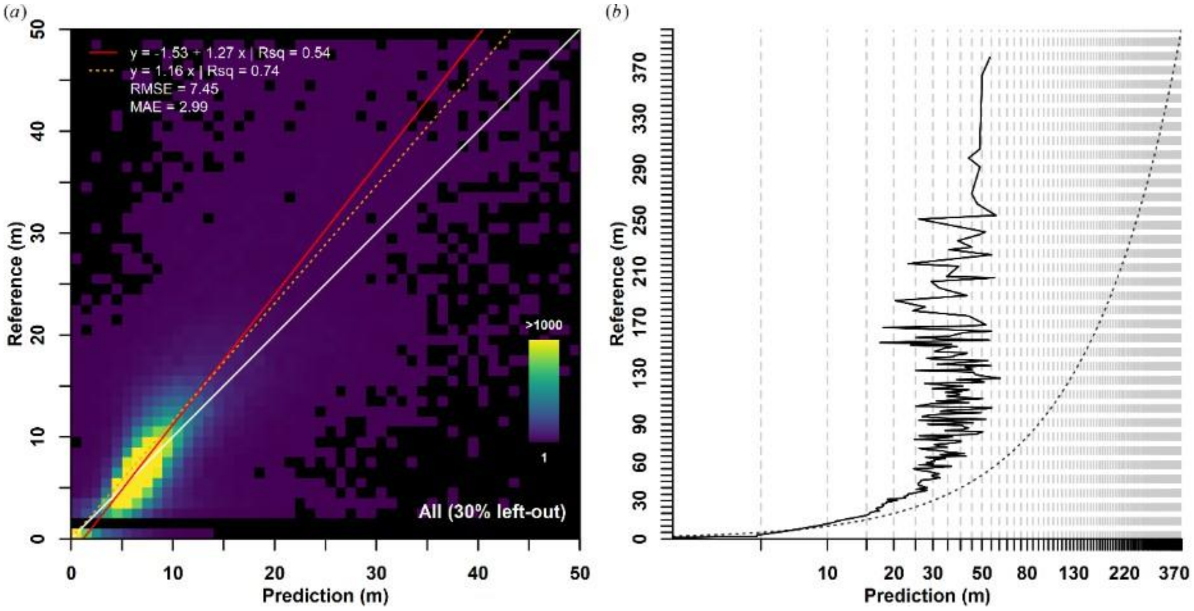

Supplementary Figure 3. Building height hold-out validation

Validation of the building height prediction with all datasets and a 70-30 data split. (**a**) density plot; white line = one-to-one; red line: ordinary least squares regression; orange line: regression through origin; RMSE = root mean squared error; MAE: mean absolute error. (**b**) median predicted building height per building height class in the reference dataset (in 1 m increments). The x-axis is drawn logarithmic and the dashed curve represents the one-to-one line.

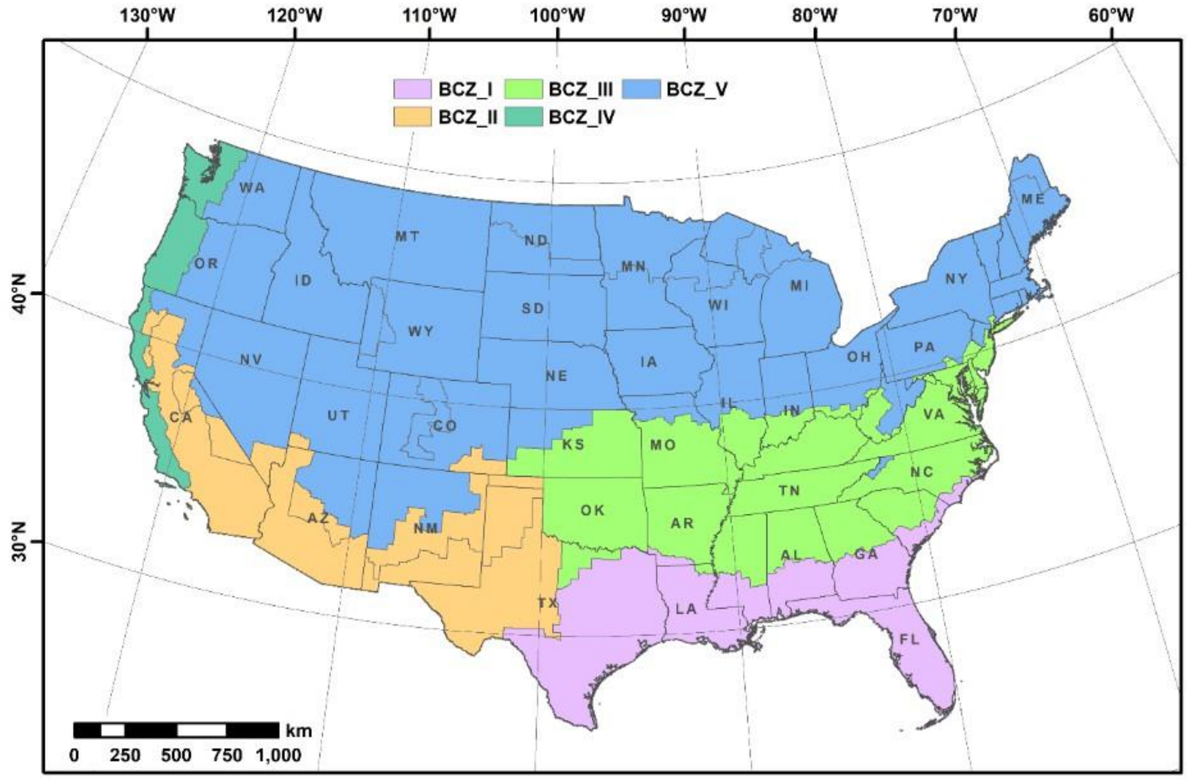

Supplementary Figure 4. Building climate zones

Climate zones are defined in Supplementary Table 8. Climate zone polygon data downloaded from https://atlas.eia.gov/maps/0c432b67293048b6a4704232a26ca99f on 22.02.2021. State boundaries were provided by the US Census Bureau.

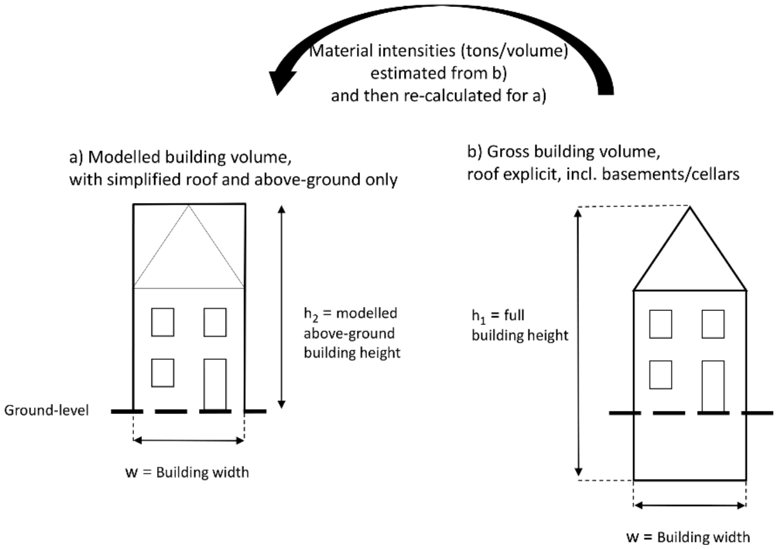

Supplementary Figure 5. Building volume definition and material factor unit conversion

The relation between common building definitions of gross building volume, and the definition of above-ground modelled building volume as used for this study. The figure was reproduced with no changes from Haberl et al. (2021), supplemental material.

Haberl, H., Wiedenhofer, D., Schug, F., Frantz, D., Virág, D., Plutzar, C., Gruhler, K., Lederer, J., Schiller, G., Fishman, T., Lanau, M., Gattringer, A., Kemper, T., Liu, G., Tanikawa, H., van der Linden, S. & Hostert, P. High-Resolution Maps of Material Stocks in Buildings and Infrastructures in Austria and Germany. Environ Sci Technol 55, 3368–3379 (2021). DOI: https://doi.org/10.1021/acs.est.0c05642.
Licensed under CC-BY 4.0 (https://creativecommons.org/licenses/by/4.0/)

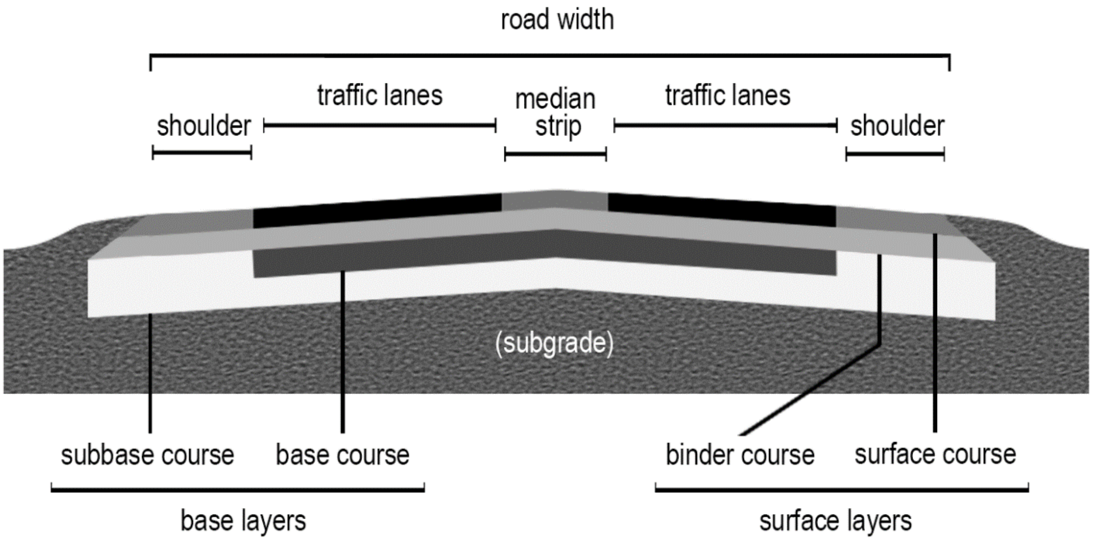

Supplementary Figure 6. Road definition

Road cross-section elements as defined in this study. In general, there are two types of pavement layers in the cross-section of paved roads: surface layers (surface and binder course) and base layers (base and subbase course). Base layers are usually made up of aggregate while either the surface is made of asphalt, concrete, or a combination thereof referred to as composite roads. The road subgrade consists of compacted local earth and is not included in the definition of socio-economic material stocks.

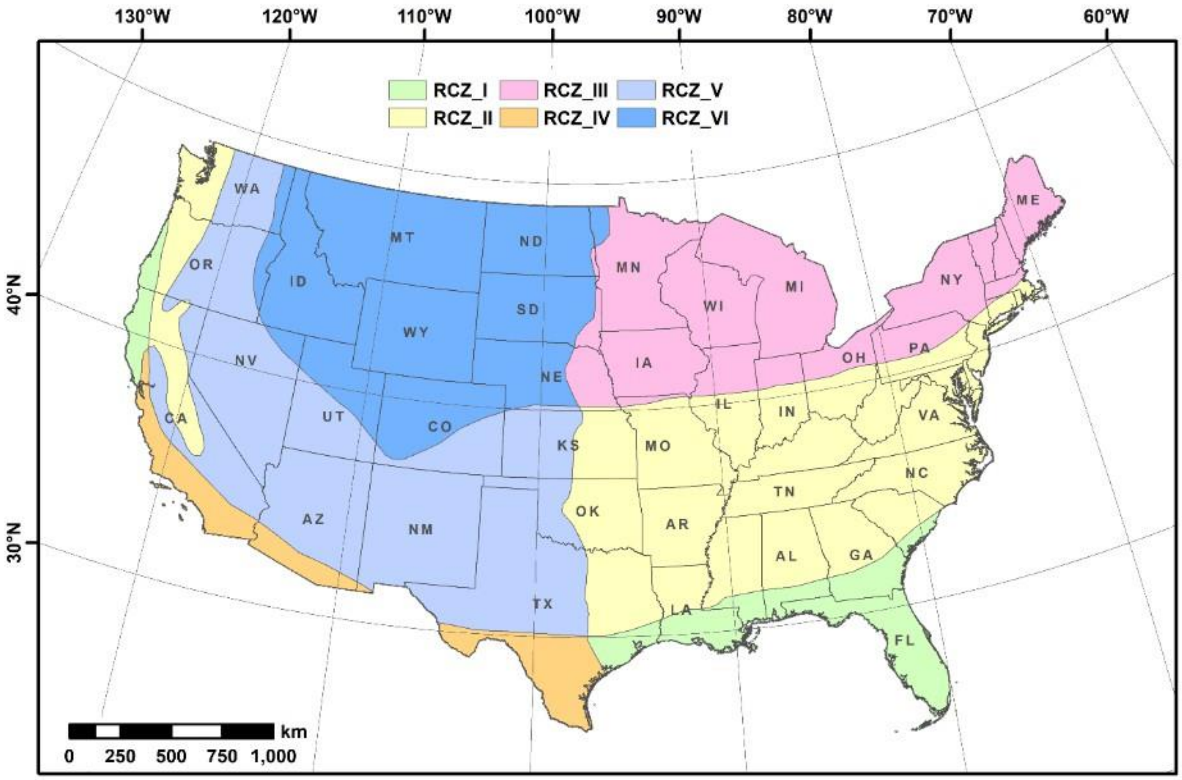

Supplementary Figure 7. Road climate zones

Road climate zones after [34] used to apply region-specific material factors for gravel roads. The map depicted in [34] was hand-digitized. For each road climate zone, mass factors for sand and gravel (aggregate) were produced based on averages of various roadbed soil qualities and equivalent single axle load traffic levels. Climate zones are defined in Supplementary Table 12. State boundaries were provided by the US Census Bureau.

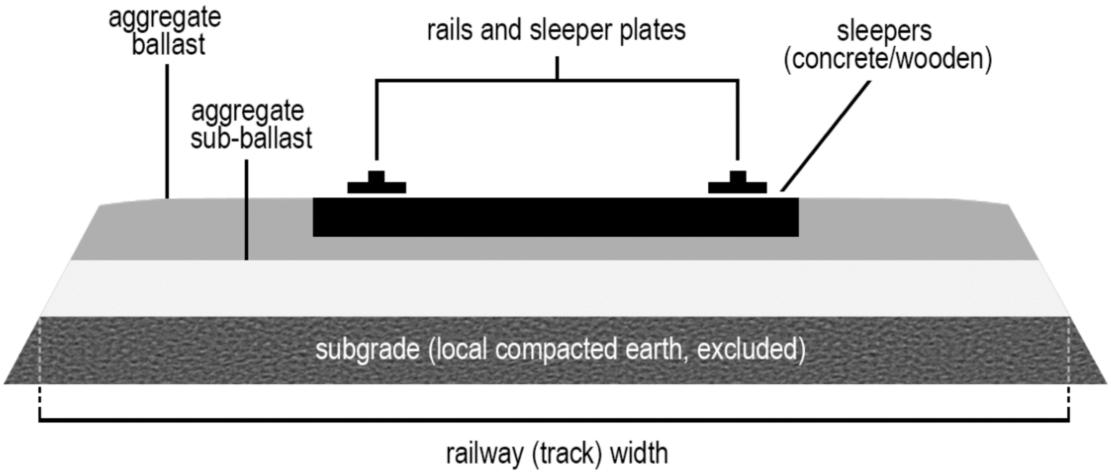

Supplementary Figure 8. Railway definition

Railway cross-section elements as defined in this study. The *Manual for Railway Engineering* [35] reports a minimum cross-section for an average single-track railway (one direction or lane), starting from steel rails, connected to wooden or concrete sleepers via sleeper plates, embedded in an aggregate ballast layer. Below this ballast layer lies a wider layer of sub-ballast. Below the sub-ballast lies the so-called subgrade, which usually consists of bulldozed and compacted local earth. In line with previous studies on railway material stocks [26,36,37], this last subgrade layer of compacted local earth is not included in the definition used herein, while the entire construction of sub-ballast, ballast layers, sleeper plates and steel rails is counted as socio-economic material stocks.

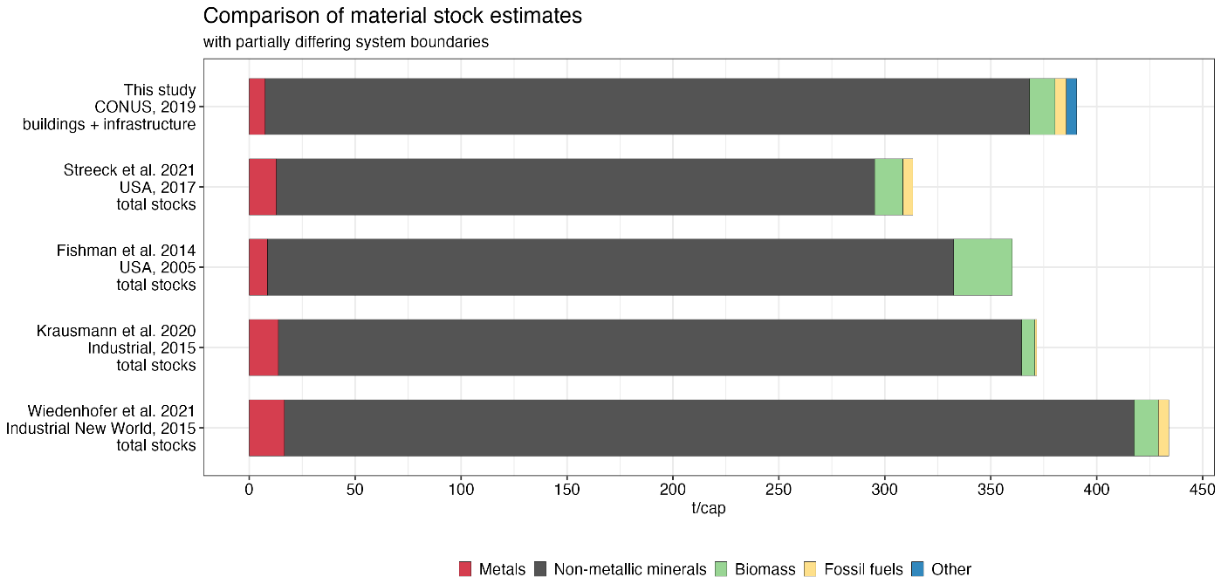

Supplementary Figure 9. Intercomparison of total mapped material stocks

Comparison of estimated per capita stocks results with those of inflow-driven studies [38–41]. Note that other studies refer to total material stocks that include stock types beyond the scope of this paper, e.g., machinery. Also note that only two studies estimate the material stock of the United States explicitly while the other studies refer to per capita stocks of Industrial countries and Industrial New World countries respectively.

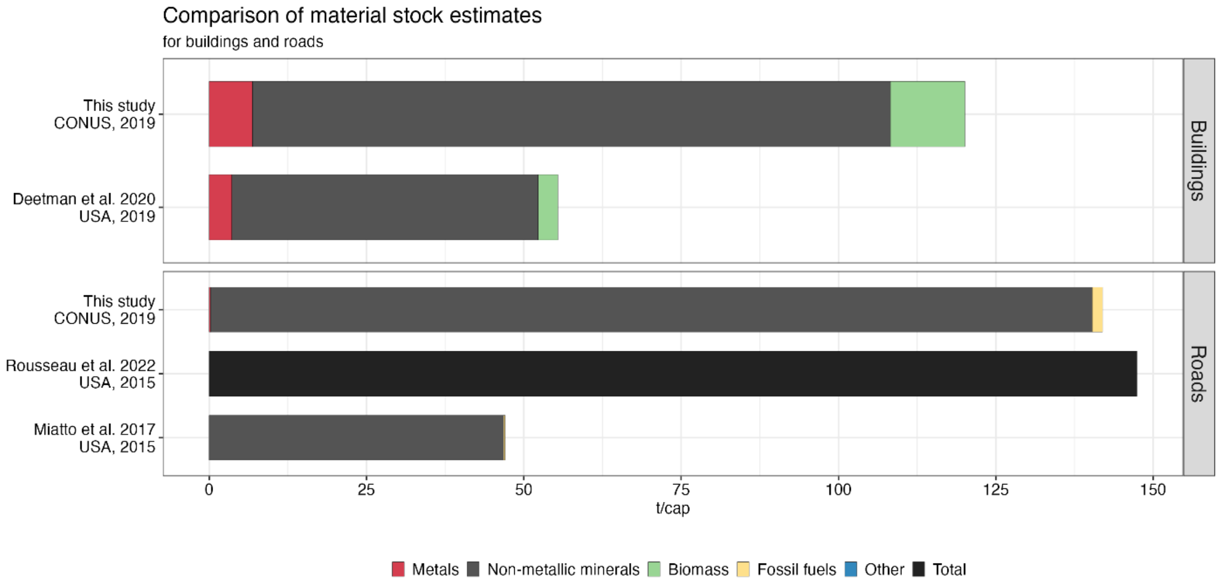

Supplementary Figure 10. Intercomparison of mapped stocks in buildings and roads

Comparison of estimated per capita road and building stocks results with those of other bottom-up studies [42,43]. The road stocks comparison excludes bridges and tunnels. Note that for the sake of comparison, results for this study were trimmed to only those material categories included in the respective comparison study. This study's material stocks estimates are significantly higher than those of some comparison studies. In the case of roads, this can in part be explained by Miatto et al. [42] using lower material intensity factors and the use of official network extent statistics which cover significantly fewer road kilometers compared to OSM data as used in this study. Likewise, material intensity factors used for buildings in Deetman et al. [43] may differ to ours and bottom-up modelling via statistical data can be expected to yield lower stocks estimates. In addition, Deetman et al. [43] did not include some building types included in this study, such as industrial buildings or light-weight structures. Rousseau et al. 2022, using a similar approach to this study, yield very similar total material road stocks.

Supplementary Table 1. Predictive variables building height

Earth Observation variables from Sentinel-1[*] and Sentinel-2 used for modelling building height; the method for selecting these features is documented in the supplemental material of [1]. Aggregation percentages represent percentiles.

Band or index	Aggregation statistic	Texture metric
Vertical-horizontal (Sentinel-1 VH)	Average	Opening
Red (Sentinel-2 band 4)	90%	Erosion
Near Infrared (Sentinel-2 band 8A)	100%	Blackhat
Tasseled Cap Greenness [44]	50%	Closing
Vertical-vertical (Sentinel-1 VV)	10%	Opening
Red edge 3 (Sentinel-2 band 7)	75%	Erosion
Near Infrared (Sentinel-2 band 8A)	90%	Erosion
Vertical-vertical (Sentinel-1 VV)	Average	Closing
Blue (Sentinel-2 band 2)	0%	Gradient
Green (Sentinel-2 band 3)	0%	Closing
Tasseled Cap Greenness [44]	Average	Opening
Tasseled Cap Greenness [44]	0%	Closing
Red edge 2 (Sentinel-2 band 6)	75%	Erosion
Red edge 2 (Sentinel-2 band 6)	90%	Opening
Blue (Sentinel-2 band 2)	25%	Closing
Tasseled Cap Brightness [44]	25%	Erosion
Near Infrared (Sentinel-2 band 8)	Average	Opening
Red edge 3 (Sentinel-2 band 7)	Average	Erosion
Green (Sentinel-2 band 3)	Average	Erosion
Red edge 2 (Sentinel-2 band 6)	10%	Opening
Near Infrared (Sentinel-2 band 8)	10%	Opening
Vertical-vertical (Sentinel-1 VV)	75%	Closing
Vertical-vertical (Sentinel-1 VV)	75%	Closing
Modified Normalized Difference Water Index [45]	25%	Gradient
Vertical-vertical (Sentinel-1 VV)	Kurtosis	Opening
Vertical-horizontal (Sentinel-1 VH)	Average	Closing
Vertical-horizontal (Sentinel-1 VH)	25%	Dilation
Blue (Sentinel-2 band 2)	0%	Dilation
Near Infrared (Sentinel-2 band 8)	90%	Opening
Blue (Sentinel-2 band 2)	25%	Dilation
Tasseled Cap Greenness [44]	25%	Erosion
Near Infrared (Sentinel-2 band 8A)	75%	Erosion
Red edge 2 (Sentinel-2 band 6)	10%	Erosion
Vertical-horizontal (Sentinel-1 VH)	Average	Dilation
Vertical-vertical (Sentinel-1 VV)	Kurtosis	Opening
Vertical-horizontal (Sentinel-1 VH)	10%	Closing
Vertical-vertical (Sentinel-1 VV)	0%	Dilation
Near Infrared (Sentinel-2 band 8)	0%	Erosion
Near Infrared (Sentinel-2 band 8A)	90%	Gradient
Red edge 2 (Sentinel-2 band 6)	Range	Opening
Vertical-horizontal (Sentinel-1 VH)	IQR	Dilation
Vertical-vertical (Sentinel-1 VV)	Kurtosis	Opening
Modified Normalized Difference Water Index [45]	25%	Gradient
Near Infrared (Sentinel-2 band 8)	10%	Erosion
Green (Sentinel-2 band 3)	0%	Closing
Green (Sentinel-2 band 3)	Average	Opening
Vertical-horizontal (Sentinel-1 VH)	25%	Closing

Red edge 2 (Sentinel-2 band 6)	100%	Erosion
Red (Sentinel-2 band 4)	75%	Closing
Green (Sentinel-2 band 3)	0%	Gradient

[*] As some data gaps were apparent in the Sentinel-1 dataset, we additionally trained a fallback model that only considered independent variables originating from Sentinel-2. Tests in [1] have shown that a model with optical data only can achieve performances comparable to a model trained with both optical and radar data. The fallback model was only applied to the few areas with limited Sentinel-1 coverage and results were checked for consistency, especially at the edge of the cutline. No validation data was available within this region.

Supplementary Table 2. Predictive variables building types

Earth Observation variables used for modelling building type with Random Forest classification following [26,46], here supplemented by a building centroid density variable to better distinguish between small-scale buildings against large commercial, industrial and office buildings that spectrally resemble, but are less densely packed.

**Band or index**	**Aggregation statistic**	**Texture metric**	**Number of features**
Vertical-horizontal (Sentinel-1 VH)	median	opening, closing	2
Blue	median, IQR	opening, closing	4
Green (Sentinel-2 band 3)	median, IQR	opening, closing	4
Red (Sentinel-2 band 4)	median, IQR	opening, closing	4
Red Edge 1	median, IQR	opening, closing	4
Near Infrared (Sentinel-2 band 8A)	median, IQR	opening, closing	4
Near Infrared (Sentinel-2 band 8)	median, IQR	opening, closing	4
Shortwave Infrared 1 (Sentinel-2 band 11)	median, IQR	opening, closing	4
Shortwave Infrared 2 (Sentinel-2 band 12)	median, IQR	opening, closing	4
Tasseled Cap Greenness [44]	median	opening, closing	2
Modified Normalized Difference Water Index [45]	median, IQR	opening, closing	4
Building centroids (Microsoft building footprints)	none	count	1

Supplementary Table 3. Building height cross-validation and data sources

Cross-validation of building height prediction. OLS: ordinary least squares regression; RTO: regression through origin; RMSE = root mean squared error; MAE: mean absolute error.

Site	OLS			RTO		RMSE	MAE	Source, date downloaded
	offset	slope	R^2	slope	R^2			
Albuquerque	-0.58	0.90	0.57	0.82	0.87	2.59	1.87	https://hub.arcgis.com/datasets/e65e375b680345e0b21fa7585d83ce9c_0, 26.10.2020
Arlington	+0.90	0.66	0.56	0.76	0.96	2.40	1.99	https://hub.arcgis.com/search?q=arlington%20building, 26.10.2020
Austin	-1.08	1.52	0.48	1.39	0.81	6.03	3.47	https://data.austintexas.gov/Locations-and-Maps/Building-Footprints-Year-2013/7bns-7teg, 26.10.2020
Boston	-1.64	1.19	0.40	1.09	0.69	10.16	3.70	http://www.bostonplans.org/3d-data-maps/3d-smart-model, 27.10.2020
Boulder	+2.04	0.75	0.46	1.03	0.89	2.43	1.66	https://hub.arcgis.com/datasets/0d43652d038a4a0dbca68f0501151bb0_0, 26.10.2020
Cambridge	+0.00	1.12	0.54	1.12	0.82	7.39	3.66	https://www.cambridgema.gov/GIS/gisdatadictionary/Basemap/BASEMAP_Buildings, 28.10.2020
Cincinnati	-2.89	1.56	0.50	1.25	0.81	5.59	2.95	https://hub.arcgis.com/datasets/CAGISPortal::building-footprints, 26.10.2020
DC	-0.46	1.15	0.41	1.11	0.81	6.63	3.90	https://app.box.com/s/zthxhdiajnkr6ncim6buahoqf5jqtksm, 28.10.2020
Englewood	+1.41	0.67	0.47	0.86	0.90	2.11	1.30	https://hub.arcgis.com/datasets/englewoodgov::building-footprints, 26.10.2020
Fort Collins	+1.42	0.76	0.52	0.98	0.89	1.97	1.32	https://hub.arcgis.com/datasets/7e577a14c83f4d83a6b58657c48027da_0, 26.10.2020
Hayward	+0.85	0.61	0.54	0.71	0.90	2.64	2.02	https://hub.arcgis.com/datasets/Hayward::hayward-building-footprints, 26.10.2020
Henderson	+0.77	0.77	0.47	0.86	0.90	2.50	1.74	https://hub.arcgis.com/datasets/23e5f3506f034c3d99b84e54fce51584_11, 26.10.2020
Lincoln	-0.46	1.08	0.48	1.03	0.87	3.24	2.08	https://hub.arcgis.com/datasets/1b6a5a2ef1b34c28950c4e720e8d7a3d_0, 26.10.2020
Los Angeles	-1.57	1.68	0.52	1.55	0.75	10.39	5.02	https://egis-lacounty.hub.arcgis.com/datasets/countywide-building-outlines-2014, 29.10.2020
Miami	-4.96	2.18	0.48	1.69	0.63	12.26	4.76	https://mdc.maps.arcgis.com/sharing/rest/content/items/ab4d3a61e60c441bbfc1098d701fc991, 28.10.2020
New York	-2.42	1.10	0.34	1.00	0.68	14.87	7.49	https://data.cityofnewyork.us/Housing-Development/Building-Footprints/nqwf-w8eh, 28.10.2020
Newport News	+2.66	0.48	0.19	0.76	0.84	3.70	2.76	https://hub.arcgis.com/datasets/nngov::building-footprints, 27.10.2020
Norman	+0.82	0.92	0.56	1.03	0.87	2.45	1.61	https://hub.arcgis.com/datasets/d68b0defa057465db7167d9260c90ad9_0, 26.10.2020
Peoria	+2.99	1.10	0.30	1.52	0.78	5.98	4.14	https://hub.arcgis.com/datasets/peoriacountygis::building-outlines, 26.10.2020
Philadelphia	-6.26	1.82	0.39	1.58	0.67	29.03	15.40	https://www.pasda.psu.edu/uci/DataSummary.aspx?dataset=7145, 10.11.2020
Portland	-0.31	0.95	0.60	0.92	0.83	3.71	2.11	https://hub.arcgis.com/datasets/PDX::building-footprints, 28.10.2020
Roanoke	+1.09	0.90	0.51	1.04	0.87	2.81	1.87	https://hub.arcgis.com/datasets/198c95ddd5f749ca9fc851dd64ba6ff0_32, 26.10.2020
Salt Lake	+2.19	0.40	0.09	0.71	0.84	2.86	2.23	https://hub.arcgis.com/datasets/slco::salt-lake-county-building-footprints, 26.10.2020
San Francisco	-1.74	1.39	0.49	1.27	0.76	9.90	4.18	https://data.sfgov.org/Geographic-Locations-and-Boundaries/Building-Footprints/ynuv-fyni, 27.10.2020
Santa Clara	+1.50	0.65	0.39	0.82	0.85	3.07	2.02	https://hub.arcgis.com/datasets/ee83a3518a7249fda22866117463de3f_0, 26.10.2020
Sarasota	-0.32	0.73	0.43	0.69	0.75	3.99	2.91	https://hub.arcgis.com/datasets/6c679d2949544274aee3bee8182c5611_0, 26.10.2020
Sauk County	+0.85	0.72	0.40	0.85	0.79	2.60	1.86	https://hub.arcgis.com/datasets/dbe64a71897e4982934dbd7637d576d5_0, 26.10.2020
Sioux Falls	+0.88	0.78	0.35	0.88	0.84	3.14	2.09	https://hub.arcgis.com/datasets/065e40f79b784848b403130234d95a1e_5, 20.10.2020
Southeast Michigan	-1.83	1.24	0.51	1.07	0.74	6.01	2.76	https://maps-semcog.opendata.arcgis.com/datasets/building-footprints-1, 28.10.2020
St Augustine	-0.36	0.99	0.61	0.94	0.85	2.39	1.54	https://hub.arcgis.com/datasets/STAUG::buildingfootprints, 26.10.2020
Tempe	+1.54	1.12	0.43	1.31	0.85	4.64	3.15	https://hub.arcgis.com/datasets/tempegov::building-footprints-usgs, 26.10.2020
**Average**	**-0.16**	**1.03**	**0.45**	**1.05**	**0.81**	**5.78**	**3.21**	Average of all lines above

Supplementary Table 4. Building types

Definitions of building types as used in this study.

Building type code	Building type name	Description
RES-LR	low-rise residential	(Semi-) Detached residential structure and attached small-scale residential or (rarely) commercial structure with a height lower than 10 m*
RES-MR	mid-rise residential	(Semi-) Detached residential structure and attached small-scale residential or (rarely) commercial structure with a height between 10 and 30 m*
RCMU	low/mid-rise residential / commercial mixed-use	Detached and attached medium to large residential, commercial or office structure lower than 30 m*
RCMU-HR	high-rise residential / commercial mixed-use	Detached and attached medium to large residential, commercial or office structure between 30 and 75 m*
RCMU-SKY	residential / commercial mixed-use skyscrapers	Detached and attached medium to large residential, commercial or office structure higher than 75 m (includes skyscrapers)*
C/I	commercial / industrial	Attached or detached light small-scale commercial, industrial or office structure and large industrial or retail and heavy industry
MLB	mobile homes + lightweight	Mobile homes and detached light-weight buildings such as wooden cabins, huts, garages, etc.

* Intermediate building types (Table S5) were translated into relevant building types from a material-specific point of view. Buildings lower than 2 m were labeled as no building, which reduced overdetection in the building footprint data and accounted for temporal misalignments between our employed EO data and the Bing imagery used by Microsoft. Buildings taller than 75 m were labeled as skyscrapers, and buildings between 30 and 75 m were labeled as "high-rise residential/commercial mixed use". The residential buildings were further split into low- and mid-rise buildings using a 10 m threshold, i.e., approximately three stories. Following the procedure in [26], we further reduced the building area of the lowrise residential buildings by 10% and added this share to the lightweight buildings category to represent attached garages and other accessory buildings that cannot be reliably separated using EO data.

Supplementary Table 5. Intermediate building types

Building type classes for Earth Observation-based mapping.

Mapping level classes	Code	Sampling level classes
Residential Buildings	RES	(Semi-) Detached residential buildings
		Attached residential buildings
Commercial and Industrial Buildings	CI	Small commercial / office building including airport buildings
		Large commercial / (heavy) industrial building
Residential / Commercial Mixed-Use Buildings	RCMU	Medium to large commercial / mixed-use buildings, mostly inner-city
Light-Weight Buildings	MLB	Lightweight buildings (e.g., sheds, mobile homes)

Supplementary Table 6. Building type training sites

Training sites for building type prediction. The sites were equally distributed within the nine U.S. Geographic Regions identified by the *National Centers for Environmental Information* [47], accounting for possibly different building structures across the country. Both urban agglomerations and rural settlements as well as a broad variety of bioclimatic conditions were represented within and across the sampling sites. We collected a maximum of 30 samples per class and site.

**Major City in the region**	**Geographic zone**	**Total number of samples**
Bend, OR	Northwest	105
Seattle, WA	Northwest	128
Idaho Falls, ID	Northwest	116
San Leandro, CA	West	132
Las Vegas, NV	West	137
Los Angeles, CA	West	165
Casper, WY	West North Central	119
Bismarck, ND	West North Central	118
Omaha, NE	West North Central	142
Salt Lake City, UT	Southwest	143
Phoenix, AZ	Southwest	147
Boulder, CO	Southwest	135
Des Moines, IA	East North Central	119
St. Paul, MN	East North Central	124
Detroit, MI	East North Central	122
Chicago, IL	Ohio Valley	143
Nashville, TE	Ohio Valley	111
Parkersburg, WV	Ohio Valley	100
Amarillo, TX	South	114
Dallas, TX	South	143
New Orleans, LA	South	118
Philadelphia, PA	Northeast	125
New York, NY	Northeast	135
Boston, MA	Northeast	118
Birmingham, AL	Southeast	117
Miami, FL	Southeast	116
Raleigh, NC	Southeast	103

Supplementary Table 7. Quality of building type prediction

Confusion matrix of the building type classification. See Supplementary Table 5 for the class definition. n = number of samples, OA = Overall Accuracy

		**Reference**					
		**RES**	**CI**	**RCMU**	**MLB**	**n**	**UA [%]**
**Prediction**	**RES**	261	47	5	15	328	79.57%
	**CI**	27	404	14	3	448	90.18%
	**RCMU**	4	44	91	0	139	65.47%
	**MLB**	36	8	1	56	101	55.45%
	**n**	328	503	111	76	427	
	**PA [%]**	79.57	80.32	81.98	75.68		**OA: 79.92**

Supplementary Table 8. Building climate zones

Building climate zones as of [48].

Zone	Short description	Description
BCZ_I	Hot-humid	A hot-humid climate is defined as a region that receives more than 50 cm of annual precipitation and where one or both of the following occur:<br>• a 19.5°C or higher wet bulb temperature for 3,000 or more hours during the warmest six consecutive months of the year; or<br>• a 23°C or higher wet bulb temperature for 1,500 or more hours during the warmest six consecutive months of the year.
BCZ_II	Hot-dry / mixed-dry	A hot-dry climate is defined as a region that receives less than 50 cm of annual precipitation and where the monthly average outdoor temperature remains above 7°C throughout the year.<br>A mixed-dry climate is defined as a region that receives less than 50 cm of annual precipitation, has approximately 5,400 heating degree days (18°C basis) or less, and where the average monthly outdoor temperature drops below 7°C during the winter months.
BCZ_III	Mixed-humid	A mixed-humid climate is defined as a region that receives more than 50 cm of annual precipitation, has approximately 5,400 heating degree days (18°C basis) or fewer, and where the average monthly outdoor temperature drops below 7°C during the winter months.
BCZ_IV	Marine	A marine climate is defined as a region that meets all of the following criteria:<br>• a coldest month mean temperature between -3°C and 18°C<br>• a warmest month mean of less than 22°C<br>• at least 4 months with mean temperatures higher than 10°C<br>• a dry season in summer. The month with the heaviest precipitation in the cold season has at least three times as much precipitation as the month with the least precipitation in the rest of the year. The cold season is October through March.
BCZ_V	Cold / very cold	A cold climate is defined as a region with between 5,400 and 9,000 heating degree days (18°C basis).<br>A very cold climate is defined as a region with between 9,000 and 12,600 heating degree days (18°C basis).

Supplementary Table 9. Mass factors for buildings

Mass factors per building type and main material category in [kg/m³]. Totals may not add up due to rounding. In total, mass factors for 15 material sub-categories were developed and used in this study. We published the full mass-per-volume factor dataset as supplemental data to this article [28].

Building type	Climate zone	mass factor (kg/m^3)						Number of case study buildings		References
		Metals	Non-metallic minerals	Biomass-based	Petrochem.-based	Others	Total	USA	other	
**RES-LR**	BCZ_I	3.7	148.5	20.6	9.8	14.2	196.9	3	0	[49,50]
	BCZ_II	2.8	161.8	25.4	5.5	7.1	202.7	7	0	[49,51,52]
	BCZ_III	6.1	250.7	29.4	7.2	10.6	304.1	5	4	[49,50,53–56]
	BCZ_IV	6.5	185.3	15.6	7.1	14.5	228.8	3	3	[49,50,53,55]
	BCZ_V	3.6	299.0	28.2	4.4	8.5	343.7	7	6	[49–51,57–59]
RES-MR<br>RCMU	All zones	13.3	277.3	19.9	0.6	3.6	314.7	8	7	[50–53,55,59–62]
RCMU-HR		18.0	255.9	3.4	0.9	0.3	278.5	2	2	[51,63–65]
RCMU-SKY		33.7	312.5	0.0	0.0	0.2	346.5	0	6	[66–68]
C/I		17.6	259.2	0.2	0.9	3.0	280.8	11	1	[52,59,69–71]
MLB		13.9	114.8	14.8	2.7	5.1	151.3	1	1	[33,50]

Supplementary Table 10. Road types and buffer widths

Definitions and re-classification of roads as given in OpenStreetMap to road types as used in this study based on shared characteristics such as pavement thickness, surface, and road width. Buffer widths span half the entire road width, including shoulders and median strip, if present (see Supplementary Figure 6). We additionally classified motorways and motorway links on bridges, as well as all other roads on bridges if the bridge attribute of a feature was set. We also differentiated bridges and tunnels, excluding the roads in and on them. Since local roads and rural roads vary in the number of lanes, an average number of 1.5 lanes was assumed for both, based on design manuals, and selective screening of Google Earth images. To account for the diversity in road design standards across the CONUS, the averages of officially reported road widths [72–74] and various county-level road design manuals were used for the individual road classes. When transforming buffered polygons to image-based area, it was made sure that the sum of all road raster layers did not exceed a completely filled pixel. In case this did happen, roads were prioritized from top to bottom; Tunnels and bridges were not subject to this condition.

Road type	OSM class	OSM class definition	Buffer width (m)
Motorway	*motorway*	A restricted access major divided highway, normally with 2 or more running lanes plus emergency hard shoulder. Equivalent to the Freeway, Autobahn, etc.	13.6
	motorway_link	The link roads (sliproads/ramps) leading to/from a motorway from/to a motorway or lower class highway. Normally with the same motorway restrictions.	6.5
Primary	*trunk*	The most important roads in a country's system that aren't motorways. (Need not necessarily be a divided highway.)	9.6
	trunk_link	The link roads (sliproads/ramps) leading to/from a trunk road from/to a trunk road or lower class highway.	6.5
Secondary	*primary*	The next most important roads in a country's system (often link larger towns).	6.0
	primary_link	The link roads (sliproads/ramps) leading to/from a primary road from/to a primary road or lower class highway.	5.5
Tertiary	*secondary*	The next most important roads in a country's system. (Often link towns.)	5.3
	secondary_link	The link roads (sliproads/ramps) leading to/from a secondary road from/to a secondary road or lower class highway.	5.1
Local	*tertiary*	The next most important roads in a country's system (often link smaller towns and villages).	4.9
	tertiary_link	The link roads (sliproads/ramps) leading to/from a tertiary road from/to a tertiary road or lower class highway.	4.5
	unclassified	The least important through roads in a country's system – i.e., minor roads of a lower classification than tertiary, but which serve a purpose other than access to properties (often link villages and hamlets).	4.5
	residential	Roads which serve as an access to housing, without function of connecting settlements. Often lined with housing.	4.5
	living_street	For living streets, which are residential streets where pedestrians have legal priority over cars, speeds are kept very low and where children are allowed to play on the street.	4.5
	service	For access roads to, or within an industrial estate, camp site, business park, car park, alleys, etc.	2.5
	footway	For designated footpaths; i.e., mainly/exclusively for pedestrians. This includes walking tracks and gravel paths.	1.8
	cycleway	For designated cycleways.	1.5
	steps	For flights of steps (stairs) on footways.	1.5

	pedestrian	For roads used mainly/exclusively for pedestrians in shopping and some residential areas.	4.0
	rest_area	Place where drivers can leave the road to rest, but not refuel.	6.0
	platform	A platform at a bus stop or station.	1.8
	services	A service station to get food and eat something, often found at motorways.	6.0
	road	A road/way/street/motorway/etc. of unknown type. It can stand for anything ranging from a footpath to a motorway.	4.0
Rural	*track_1*	Roads for mostly agricultural or forestry uses. Solid. Usually a paved or sealed surface.	2.5
	track_2	Roads for mostly agricultural or forestry uses. Solid but unpaved. Usually an unpaved track with surface of gravel.	2.5
	track_3	Roads for mostly agricultural or forestry uses. Mostly solid. Even mixture of hard and soft materials. Almost always an unpaved track.	2.0
	track_4	Roads for mostly agricultural or forestry uses. Mostly soft. Almost always an unpaved track prominently with soil/sand/grass, but with some hard or compacted materials mixed in.	2.0
	track_5	Roads for mostly agricultural or forestry uses. Soft. Almost always an unimproved track lacking hard materials, same as surrounding soil.	2.0
	track_na	Roads for mostly agricultural or forestry uses. No tracktype tag present.	2.0

Supplementary Table 11. Mass factors for roads

Mass factors and widths for road types. See Supplementary Table 12 for the climate zones. Pavement type describes the type of surface that was factored in for the calculation of mass-per-area factors. Reported pavement layer thicknesses were multiplied by standard material densities (sand and gravel: 2.71 t m^{-3}, asphalt: 2.73 t m^{-3}, concrete: 2.41 t m^{-3}) [26,75], thus providing the mass of material per square meter of road (t m^{-2}) per road type. Asphalt was further split into its material components bitumen and aggregate with a 5:95 ratio [26,75].We published the full MI data as supplemental data to this article in [28].

**Road type**	**Climate zone**	**Pavement type**	**Material intensity (kg m^{-2})**					**Width (m)**	**References for**	
			Steel	Concrete	Aggregate	Bitumen	Total		MI	Width
Motorway	N/A	Flexible	-	215.2	991.0	12.3	1,218.5	27.2	[76]	[72,73,77–79]
Primary		Rigid	-	115.7	934.7	14.8	1,065.2	19.3	[76,77,80–83]	[72,73,78,79,84]
Secondary		Composite	-	76.0	879.6	13.8	969.3	12.1	[74,76,77,80–83,85–90]	[72–74,77–79,84,91]
Tertiary			-	40.5	772.1	11.5	824.1	10.7		
Local	RCZ_I	Flexible	-	7.0	325.0	3.8	335.9	7.7		
	RCZ_I	Rigid	-	7.0	328.5	3.8	339.4			
	RCZ_II	Composite	-	7.0	334.5	3.8	345.4			
	RCZ_V	Gravel	-	7.0	322.4	3.8	333.2			
	RCZ_V	Dirt	-	7.0	325.2	3.8	336.1			
	RCZ_VI		-	7.0	333.6	3.8	344.5			
Rural	RCZ_I	Flexible	-	0.5	114.5	0.3	115.3	6.2	[74]	[72,90]
	RCZ_II	Rigid	-	0.5	122.0	0.3	122.8			
	RCZ_III	Composite	-	0.5	134.9	0.3	135.7			
	RCZ_V	Gravel	-	0.5	108.7	0.3	109.5			
	RCZ_V	Dirt	-	0.5	114.8	0.3	115.7			
	RCZ_VI		-	0.5	132.8	0.3	133.7			
Motorways on bridges [1]	N/A	Same as road type	-	-	417.3	22.0	439.2	Same as road type	[36,37,75,92]	Same as road type
Other roads on bridges [1]			-	-	286.8	15.1	301.9			
Motorway bridges [2]			116.8	1,534.0	-	-	1,650.7		[93]	
Other road bridges [2]			136.0	1,295.3	-	-	1,431.3			
Tunnels [3]			171.8	4,556.7	-	-	4,728.5		[36]	

[1] Excluding bridge structure; [2] Excluding road surface; [3] Unit: kg m^{-2} tube

Supplementary Table 12. Road climate zones

Road climate zones after [34].

Zone	Description
**RCZ_I**	Wet, no freeze
**RCZ_II**	Wet, freeze-thaw cycle
**RCZ_III**	Wet, hard-freeze, spring thaw
**RCZ_IV**	Dry, no freeze
**RCZ_V**	Dry, freeze-thaw cycle
**RCZ_VI**	Dry, hard freeze, spring thaw

Supplementary Table 13. Railway types and buffer widths

Definitions and re-classification of railways as given in OpenStreetMap to railway types as used in this study based on functional characteristics. We additionally separated bridges and tunnels, excluding the rails in and on them if the bridge attribute of a feature was set. Subways were further split into underground tracks, aboveground tracks, and elevated tracks. Buffer widths span half of the entire railway width. Width estimates were adopted from [26] with the underlying assumption that US-American and Austrian/German track widths are comparable, knowing that all three countries use international gauge. When transforming buffered polygons to image-based area, it was made sure that the sum of all raster layers did not exceed a completely filled pixel. In case this did happen, roads were prioritized from top to bottom; Tunnels and bridges were not subject to this condition.

Railway type	OSM class	OSM class definition	Buffer width (m)
Railway	*rail*	Full sized passenger or freight trains in the standard gauge for the country or state.	6.0
	light_rail	A higher-standard tram system, normally in its own right-of-way. Often it connects towns and thus reaches a considerable length (tens of kilometers).	3.5
Subway	*subway*	A city passenger rail service running mostly grade separated. Often a significant portion of the line or its system/network is underground.	5.0
Tram	*tram*	One or two carriage rail vehicles, usually sharing motor road, sometimes called "street running".	3.5
Other	*narrow_gauge*	Narrow-gauge passenger or freight trains. Narrow-gauge railways can have mainline railway service like the Rhaetian Railway in Switzerland or can be a small light industrial railway.	3.5
	preserved	A railway running historic trains, usually a tourist attraction.	3.5
	disused	A section of railway which is no longer used but where the track and infrastructure remains in place.	2.0
	funicular	Cable driven inclined railways on a steep slope, with a pair of cars connected by one cable.	3.5
	monorail	A railway with only a single rail. A monorail can run above the rail like in Las Vegas and Disneyland or can suspend below the rail like the Wuppertal Schwebebahn (Germany).	3.5
	miniature	Miniature railways are narrower than narrow gauge and carry passengers, frequently at an exact scale of "standard-sized" rail (for example "1/4 scale"). They can often be found in parks.	2.0

Supplementary Table 14. Mass factors for railways

Mass factors and widths for railway types. Totals may not add up due to rounding. For metals, we further distinguish between iron or steel, copper and aluminum. We published the full mass factor dataset as supplemental data to this article in [28].

Railway type	Further specifications	Material factor (kg m^{-2})					Width (m)	References
		Metals	Concrete	Aggregate	Timber	Total		
Railway	Above ground	17.2	5.0	807.0	24.7	853.9	12.0[1] / 7[2]	[35,94–97]
	Tunnel [3]	153.5	4,070.4	-	-	4,223.9		[36]
	Bridge (excluding rail)	1,454.8	120.3	-	-	1,575.1		
Subway	Underground	654.7	13,188.7	-	-	13,843.4	10.0	[27]
	Above ground	254.7	2,337.6	428.2	-	3,020.5		
	Subway on bridges	361.5	4,614.4	428.2	-	5,404.1		
Tram	N/A	18.1	557.1	40.0	-	615.3	7.0	[93]
Other		18.1	557.1	40.0	-	615.3		

[1] width for OSM-key “rail”; [2] width for OSM-key “light_rail”; [3] Unit: kg m^{-2} tube

Supplementary Table 15. Mass factors for airport and parking infrastructure

Mass factors and widths for airport- and parking-related mobility infrastructure. Totals may not add up due to rounding. We published the mass factor dataset as supplemental data to this article in [28].

Type	Material factor (kg m^{-2})					References for material intensity
	Steel	Concrete	Aggregate	Bitumen	Total	
Airport runways	-	215.2	991.0	12.3	1,218.5	[76]
Parking lots and yards	-	-	559.4	11.3	570.7	[98–102]

Supplementary Table 16. Composition of remaining imperviousness areas

Investigation of the composition of the remaining impervious area class.

Label	Type[1]	Percent of samples [%]
Parking lot	I	18
Industrial yard	I	25
Residential front yard	I	15.3
Residential back yard	I	9
Public areas (e.g., plazas, marketplaces)	I	2.4
Other artificial surfaces (e.g., solar panels, water edge fortification)	II	1.7
Construction sites	II	3.3
Buildings	III	3.5
Other mobility infrastructure (e.g., cycle ways, footpaths or other roads)	IV	16
Vegetation	V	1.4
Other natural surfaces	V	4.4

[1] as referred to in Supplementary Discussion 5

Supplementary Table 17. Socio-economic variables

Potential independent variables that were linked to material intensity of buildings and mobility infrastructure on the county-level. The explanatory variables were selected to represent the target year 2018 as close as possible. Note: for each model, a variable selection was performed using stepwise multivariate regression using the Bayesian Information Criterion (BIC) as selection criterion; thus not all potential explanatory variables were used in the final model. Population density is a measure of population per area, i.e. cap km^{-2}. The average domestic migration rate was calculated as the average of annual migration rates and represents a long-term measure of within-CONUS migration patterns; negative values represent emigration; positive values represent immigration. The international migration rate analogously represents movement patterns into or out of the United States. Death and birth rates only have positive values. The urban population variable describes the percentage of the population that are living in urban areas; the remainder to 100% represents the population that are living in rural areas. The real gross domestic product (GDP), normalized by population, was chosen as an economic measure..

Variable	Vintage	Source
Population density	2018	United States Census (https://www.census.gov/data/tables/time-series/demo/popest/2010s-state-total.html)
Average domestic migration rate	2000-2018	
Average International migration rate	2000-2018	
Average Birth rate	2000-2018	
Average Death rate	2000-2018	
Household size	2020	United States Census (https://data.census.gov/cedsci/table)
Urban population	2010	United States Census (https://www.census.gov/programs-surveys/geography/guidance/geo-areas/urban-rural/2010-urban-rural.html)<br><br>See Suplementary Notes 1 for more detail
Real Gross Domestic Product (GDP) in Thousands of chained (2012) dollars per capita	2018	Bureau of Economic Analysis (https://www.bea.gov/data/gdp/gdp-county-metro-and-other-areas)

Supplementary Table 18. Multivariate linear regression results for buildings

Results of the Spatial Autoregressive Model (SAR) testing various potential predictors of material intensity of buildings. The three strongest contributors are highlighted in bold. See Supplementary Methods 5 for employed methodology; see Supplementary Table 17 for a description of predictors.

Term	estimate	std. error	statistic	p-value
**(Intercept)**	-0.05	0.10	-0.48	0.63
**Urban population**	**-0.24**	0.01	-22.93	0.00
**Average birth rate**	-0.13	0.01	-12.01	0.00
**Average domestic migration rate**	-0.17	0.01	-15.71	0.00
**Average international migration rate**	-0.07	0.01	-6.94	0.00
**Household size**	**-0.18**	0.01	-19.73	0.00
**Gross Domestic Product**	**+0.24**	0.01	23.97	0.00
**Lambda**	0.92			0.00
**Moran test of the residuals**	-0.04			0.99
**Nagelkerke pseudo R^2**	0.54			
**Chi2 test statistic (ratio test)**	1957.4			0.00
**RMSE**	0.41			

Supplementary Table 19. Multivariate linear regression results for mobility infrastructure

Results of the Spatial Autoregressive Model (SAR) testing various potential predictors of material intensity of mobility infrastructure. The three strongest contributors are highlighted in bold. See Supplementary Methods 5 for employed methodology; see Supplementary Table 17 for a description of predictors.

Term	estimate	std. error	statistic	p-value
**(Intercept)**	+0.01	0.01	0.51	0.61
**Population density**	**-0.84**	0.01	-80.60	0.00
**Urban population**	-0.05	0.01	-7.51	0.00
**Average birth rate**	-0.05	0.01	-9.22	0.00
**Average death rate**	+0.08	0.01	14.77	0.00
**Average domestic migration rate**	**-0.14**	0.01	-27.73	0.00
**Gross Domestic Product**	**+0.13**	0.00	26.77	0.00
**lambda**	0.72			0.00
**Moran test statistics of the residuals**	-0.03			0.99
**Nagelkerke pseudo R^2**	1.00			
**Chi2 test statistic (ratio test)**	6664.8			0.00
**RMSE**	0.20			

Supplementary Table 20. Collection of supplementary items describing uncertainty

This table collects references to supplementary items describing uncertainty of the individual information layers or processing steps employed during our workflow.

Information Layer	Supplementary item
Material factors	Supplementary Methods 3<br>Supplementary Discussion 4
Building area	Supplementary Discussion 1
Building height	Supplementary Discussion 2<br>Supplementary Figure 3<br>Supplementary Table 3
Building type	Supplementary Discussion 3<br>Supplementary Table 7
Parking spaces	Supplementary Discussion 5<br>Supplementary Table 16

Supplementary References

1. Frantz, D. *et al.* National-scale mapping of building height using Sentinel-1 and Sentinel-2 time series. *Remote Sens. Environ.* **252**, 112128 (2021).
2. Torres, R. *et al.* GMES Sentinel-1 Mission. *Remote Sens. Environ.* **120**, 9–24 (2012).
3. Koppel, K., Zalite, K., Voormansik, K. & Jagdhuber, T. Sensitivity of Sentinel-1 backscatter to characteristics of buildings. *International Journal of Remote Sensing* **38**, 6298–6318 (2017).
4. Farr, T. G. *et al.* The Shuttle Radar Topography Mission. *Reviews of Geophysics* **45**, (2007).
5. I. Ali, S. Cao, V. Naeimi, C. Paulik & W. Wagner. Methods to Remove the Border Noise From Sentinel-1 Synthetic Aperture Radar Data: Implications and Importance For Time-Series Analysis. *IEEE J. Sel. Topics Appl. Earth Observ. and Remote Sens.* **11**, 777–786 (2018).
6. Drusch, M. *et al.* Sentinel-2: ESA's Optical High-Resolution Mission for GMES Operational Services. *Remote Sens. Environ.* **120**, 25–36 (2012).
7. Frantz, D. FORCE—Landsat + Sentinel-2 Analysis Ready Data and Beyond. *Remote Sensing* **11**, 1124 (2019).
8. Zhu, Z. & Woodcock, C. E. Object-Based Cloud and Cloud Shadow Detection in Landsat Imagery. *Remote Sens. Environ.* **118**, 83–94 (2012).
9. Zhu, Z., Wang, S. & Woodcock, C. E. Improvement and Expansion of the Fmask Algorithm: Cloud, Cloud Shadow, and Snow Detection for Landsats 4–7, 8, and Sentinel 2 Images. *Remote Sens. Environ.* **159**, 269–277 (2015).
10. Frantz, D., Röder, A., Udelhoven, T. & Schmidt, M. Enhancing the Detectability of Clouds and Their Shadows in Multitemporal Dryland Landsat Imagery: Extending Fmask. *IEEE Geosci. Remote Sens. Lett.* **12**, 1242–1246 (2015).
11. Frantz, D., Haß, E., Uhl, A., Stoffels, J. & Hill, J. Improvement of the Fmask algorithm for Sentinel-2 images: Separating clouds from bright surfaces based on parallax effects. *Remote Sens. Environ.* **215**, 471–481 (2018).
12. Frantz, D., Röder, A., Stellmes, M. & Hill, J. An Operational Radiometric Landsat Preprocessing Framework for Large-Area Time Series Applications. *IEEE Trans. Geosci. Remote Sens.* **54**, 3928–3943 (2016).

13. Tanré, D., Herman, M., Deschamps, P. Y. & de Leffe, A. Atmospheric Modeling for Space Measurements of Ground Reflectances, Including Bidirectional Properties. *Appl. Opt.* **18**, 3587–3594 (1979).

14. Buchner, J. *et al.* Land-cover change in the Caucasus Mountains since 1987 based on the topographic correction of multi-temporal Landsat composites. *Remote Sens. Environ.* **248**, 111967 (2020).

15. Roy, D., Li, Z. & Zhang, H. Adjustment of Sentinel-2 Multi-Spectral Instrument (MSI) Red-Edge Band Reflectance to Nadir BRDF Adjusted Reflectance (NBAR) and Quantification of Red-Edge Band BRDF Effects. *Remote Sensing* **9**, 1325 (2017).

16. Roy, D. P. *et al.* Examination of Sentinel-2A multi-spectral instrument (MSI) reflectance anisotropy and the suitability of a general method to normalize MSI reflectance to nadir BRDF adjusted reflectance. *Remote Sens. Environ.* **199**, 25–38 (2017).

17. Frantz, D. *et al.* Improving the Spatial Resolution of Land Surface Phenology by Fusing Medium- and Coarse-Resolution Inputs. *IEEE Trans. Geosci. Remote Sens.* **54**, 4153–4164 (2016).

18. Bauer-Marschallinger, B., Sabel, D. & Wagner, W. Optimisation of global grids for high-resolution remote sensing data. *Computers & Geosciences* **72**, 84–93 (2014).

19. Müller, H., Rufin, P., Griffiths, P., Barros Siqueira, A. J. & Hostert, P. Mining dense Landsat time series for separating cropland and pasture in a heterogeneous Brazilian savanna landscape. *Remote Sens. Environ.* **156**, 490–499 (2015).

20. Mack, B., Leinenkugel, P., Kuenzer, C. & Dech, S. A semi-automated approach for the generation of a new land use and land cover product for Germany based on Landsat time-series and Lucas in-situ data. *Remote Sensing Letters* **8**, 244–253 (2017).

21. Potapov, P., Matthew Hansen, Stephen V. Stehman, Kyle Pittman & Svetlana Turubanova. Gross forest cover loss in temperate forests: biome-wide monitoring results using MODIS and Landsat data. *APPRES* **3**, 1–23 (2009).

22. Rufin, P. *et al.* Mapping Cropping Practices on a National Scale Using Intra-Annual Landsat Time Series Binning. *Remote Sensing* **11**, 232 (2019).

23. Schug, F., Frantz, D., Okujeni, A., van der Linden, S. & Hostert, P. Mapping urban-rural gradients of settlements and vegetation at national scale using Sentinel-2 spectral-temporal metrics and regression-based unmixing with synthetic training data. *Remote Sens. Environ.* **246**, 111810 (2020).
24. M. Dalla Mura, J. A. Benediktsson, B. Waske & L. Bruzzone. Morphological Attribute Profiles for the Analysis of Very High Resolution Images. *IEEE Trans. Geosci. Remote Sens.* **48**, 3747–3762 (2010).
25. Biljecki, F. & Chow, Y. S. Global Building Morphology Indicators. *Computers, Environment and Urban Systems* **95**, 101809 (2022).
26. Haberl, H. *et al.* High-Resolution Maps of Material Stocks in Buildings and Infrastructures in Austria and Germany. *Environ Sci Technol* **55**, 3368–3379 (2021).
27. Lederer, J. *et al.* Material Flows and Stocks in the Urban Building Sector: A Case Study from Vienna for the Years 1990–2015. *Sustainability* **12**, 300 (2019).
28. Baumgart, A., Virág, D., Frantz, D., Schug, F. & Wiedenhofer, D. Material intensity factors for buildings, roads and rail-based infrastructure in the United States. *Zenodo* https://doi.org/10.5281/zenodo.8104418 (2022).
29. R Core Team. R: A Language and Environment for Statistical Computing. (2021).
30. Bivand, R. S., Pebesma, E. J., Gómez-Rubio, V. & Pebesma, E. J. *Applied spatial data analysis with R*. vol. 747248717 (Springer, 2013).
31. Heris, M. P., Foks, N. L., Bagstad, K. J., Troy, A. & Ancona, Z. H. A rasterized building footprint dataset for the United States. *Scientific Data* **7**, 207 (2020).
32. MacEachren, A. M. *et al.* Visualizing Geospatial Information Uncertainty: What We Know and What We Need to Know. *Cartography and Geographic Information Science* **32**, 139–160 (2005).
33. Atmaca, N. Life-cycle assessment of post-disaster temporary housing. *Building Research & Information* **45**, 524–538 (2017).
34. Tirado-Crovetti, M. R., Darter, M. I., Jayawickrama, P. W., Smith, R. E. & Lytton, R. L. Ode Computer Program: Mechanistic-Empirical Asphalt Concrete Overlay Design. *Transportation Research Record* **1207**, 30–38 (1988).
35. *Manual for Railway Engineering*. (American Railway Engineering and Maintenance-of-Way Association, 2010).

36. Steger, S., Fekkak, M. & Bringezu, D. S. *Materialbestand und Materialflüsse in Infrastrukturen. Meilensteinbericht des Arbeitspakets 2.3 des Projekts 'Materialeffizienz und Ressourcenschonung' (MaRess)*. 268 (2011).

37. Wiedenhofer, D., Steinberger, J. K., Eisenmenger, N. & Haas, W. Maintenance and Expansion: Modeling Material Stocks and Flows for Residential Buildings and Transportation Networks in the EU25. *Journal of Industrial Ecology* **19**, 538–551 (2015).

38. Streeck, J., Dammerer, Q., Wiedenhofer, D. & Krausmann, F. The role of socio-economic material stocks for natural resource use in the United States of America from 1870 to 2100. *Journal of Industrial Ecology* **25**, 1486–1502 (2021).

39. Fishman, T., Schandl, H., Tanikawa, H., Walker, P. & Krausmann, F. Accounting for the Material Stock of Nations. *Journal of Industrial Ecology* **18**, 407–420 (2014).

40. Krausmann, F., Wiedenhofer, D. & Haberl, H. Growing stocks of buildings, infrastructures and machinery as key challenge for compliance with climate targets. *Global Environmental Change* **61**, 102034 (2020).

41. Wiedenhofer, D. *et al.* Prospects for a saturation of humanity's resource use? An analysis of material stocks and flows in nine world regions from 1900 to 2035. *Global Environmental Change* **71**, 102410 (2021).

42. Miatto, A., Schandl, H., Wiedenhofer, D., Krausmann, F. & Tanikawa, H. Modeling material flows and stocks of the road network in the United States 1905–2015. *Resources, Conservation and Recycling* **127**, 168–178 (2017).

43. Deetman, S. *et al.* Modelling global material stocks and flows for residential and service sector buildings towards 2050. *Journal of Cleaner Production* **245**, 118658 (2020).

44. Crist, E. P. A TM Tasseled Cap equivalent transformation for reflectance factor data. *Remote Sens. Environ.* **17**, 301–306 (1985).

45. Xu, H. Modification of normalised difference water index (NDWI) to enhance open water features in remotely sensed imagery. *International Journal of Remote Sensing* **27**, 3025–3033 (2006).

46. Schug, F., Frantz, D., van der Linden, S. & Hostert, P. Gridded population mapping for Germany based on building density, height and type from Earth Observation data using census disaggregation and bottom-up estimates. *PLOS ONE* **16**, e0249044 (2021).

47. Karl, T. R. & Koss, W. J. Regional and National Monthly, Seasonal, and Annual Temperature Weighted by Area, 1895-1983. *Historical Climatology Series* **4**, 38 pp. (1984).

48. U. S. Department of Energy. *Guide to Determining Climate Regions by County*. (2015).

49. Marceau, M. L. & VanGeem, M. G. Comparison of the Life Cycle Assessments of an Insulating Concrete Form House and a Wood Frame House. *Journal of ASTM International* **3**, 1–11 (2006).

50. Berrill, P. & Hertwich, E. G. Material flows and GHG emissions from housing stock evolution in US counties, 2020–60. *Buildings and Cities* (2021) doi:10.5334/bc.126.

51. Ochsendorf, J. *et al. Methods, Impacts, and Opportunities in the Concrete Building Life Cycle*. (2011).

52. Reyna, J. L. & Chester, M. V. The Growth of Urban Building Stock: Unintended Lock-in and Embedded Environmental Effects. *Journal of Industrial Ecology* **19**, 524–537 (2015).

53. Reza, B., Sadiq, R. & Hewage, K. Emergy-based life cycle assessment (Em-LCA) of multi-unit and single-family residential buildings in Canada. *International Journal of Sustainable Built Environment* **3**, 207–224 (2014).

54. Shreve, T. Designing for Embodied Energy: An Examination of BIM integrated LCA using Residential Architecture in Rochester, NY. (Rochester Institute of Technology, 2018).

55. Stephan, A. & Athanassiadis, A. Towards a more circular construction sector: Estimating and spatialising current and future non-structural material replacement flows to maintain urban building stocks. *Resources, Conservation and Recycling* **129**, 248–262 (2018).

56. Mosteiro-Romero, M. *et al.* Relative importance of electricity sources and construction practices in residential buildings: A Swiss-US comparison of energy related life-cycle impacts. *Energy and Buildings* **68**, 620–631 (2014).

57. Blanchard, S. & Reppe, P. *Life Cycle Analysis of a Residential Home in Michigan*. (1998).

58. Keoleian, G. A., Blanchard, S. & Reppe, P. Life-Cycle Energy, Costs, and Strategies for Improving a Single-Family House. *Journal of Industrial Ecology* **4**, 135–156 (2000).

59. Mollaei, A., Ibrahim, N. & Habib, K. Estimating the construction material stocks in two Canadian cities: A case study of Kitchener and Waterloo. *Journal of Cleaner Production* **280**, 124501 (2021).

60. Aye, L., Ngo, T., Crawford, R. H., Gammampila, R. & Mendis, P. Life cycle greenhouse gas emissions and energy analysis of prefabricated reusable building modules. *Energy and Buildings* **47**, 159–168 (2012).

61. Carre, A. & Crossin, E. A comparative Life Cycle Assessment of Two Multi Storey Residential Apartment Buildings. *Forest & Wood Products Australia* **Projekt No: PRA344-1415**, (2015).

62. Kumar, V., Kasun, H. & Sadiq, R. Life Cycle Assessment Of Residential Buildings: A Case Study In Canada. *International Journal of Energy and Environmental Engineering* **9**, 1017–1025 (2015).

63. Robati, M., McCarthy, T. J. & Kokogiannakis, G. Integrated life cycle cost method for sustainable structural design by focusing on a benchmark office building in Australia. *Energy and Buildings* **166**, 525–537 (2018).

64. Chen, Z., Gu, H., Bergman, R. & Liang, S. Comparative Life-Cycle Assessment of a High-Rise Mass Timber Building with an Equivalent Reinforced Concrete Alternative Using the Athena Impact Estimator for Buildings. *Sustainability* **12**, 4708 (2020).

65. Nasab, T. J., Monavari, S. M., Jozi, S. A. & Majedi, H. Environmental impacts Analysis of High-Rise Construction in Tehran. *Journal of Materials and Environmental Science* **11**, 1642–1657 (2020).

66. Trabucco, D. Life Cycle Energy Analysis of Tall Buildings: Design Principles. in (2012).

67. Cho, H.-W., Roh, S.-G., Byun, Y.-M. & Yom, K.-S. Structural Quantity Analysis of Tall Buildings. in (Council of Tall Buildings and Urban Habitat, 2004).

68. Helal, J., Stephan, A. & Crawford, R. H. Towards a design framework for the structural systems of tall buildings that considers embodied greenhouse gas emissions. in *Structures and Architecture. Bridging the Gap and Crossing Borders* (ed. Cruz, P. J. S.) 881–888 (CRC Press, 2019).

69. Guggemos Angela, A. & Horvath, A. Comparison of Environmental Effects of Steel- and Concrete-Framed Buildings. *Journal of Infrastructure Systems* **11**, 93–101 (2005).

70. Stadel, A., Gursel, P. & Masanet, E. *Life-Cycle Evaluation of Concrete Building Construction as a Strategy for Sustainable Cities*. LBNL-5531E, 1223003 (2012).

71. Attia, S. Towards regenerative and positive impact architecture: A comparison of two net zero energy buildings. *Sustainable Cities and Society* **26**, 393–406 (2016).

72. *A Policy on Geometric Design of Highways and Streets*. (American Association of State Highway and Transportation Officials, 2001).

73. Aashto. *A Policy on Geometric Design of Highways and Streets*. (American Association of State Highway and Transportation Officials, 2011).

74. Federal Highway Administration. *Gravel Roads. Maintenance and Design Manual. South Dakota Local Transportation Assistance Program (SD LTAP)*. (2000).

75. Virág, D. *et al.* The stock-flow-service nexus of personal mobility in an urban context: Vienna, Austria. *Environmental Development* 100628 (2021) doi:10.1016/j.envdev.2021.100628.

76. County of San Diego. *Public Road Standards - March 2012 Update*. (2012).

77. County of Jefferson. *Transportation Design & Construction Manual*. (2019).

78. Loijos, A., Santero, N. & Ochsendorf, J. Life cycle climate impacts of the US concrete pavement network. *Resources, Conservation and Recycling* **72**, 76–83 (2013).

79. Transportation Association of Canada. *B.C. Supplement to TAC Geometric Design Guide for Canadian Roads*. (2019).

80. Delatte, N. J. *Concrete pavement design, construction, and performance*. (Taylor & Francis, 2008).

81. Larimer, C. *Rural Area Road Standards*. (2007).

82. Town of Castle Rock. *Transportation Design Criteria Manual*. (2018).

83. ACPA. *Design of Concrete Pavement for Streets and Roads*. (2006).

84. Richland County. *Section 5 - Road Design Standards*. (2007).

85. City of Mississauga. *Transportation and Work Standard Drawing Design Requirements Pavement and Road Base*. (2018).

86. City of Phoenix. *Street Planning and Design Guidelines*. (2009).

87. City of Huron. *Chapter 8: Street Design and Pavement Thickness*. (2014).

88. St. Johns County. *Article VI, Design Standards and Improvement Requirements*. (2020).

89. Chicago Department of Transportation. *Rules regarding construction in the public way. Under 2-102-030(L); 2-102-040; 10-20-210*. 323 (2014).

90. Asphalt Paving Association of Iowa. Chapter 4: Thickness Design. in *Asphalt Design Guide* (ed. Asphalt Paving Association of Iowa) (Asphalt Paving Association of Iowa).

91. National Cooperative Highway Research Program. *Roadway Widths for Low-Traffic-Volume Roads*. (1994).

92. Knappe, F., Reinhardt, J., Bergmann, T. & Mottschall, M. *Substitution von Primärrohstoffen im Straßen- und Wegebau durch mineralische Abfälle und Bodenaushub; Stoffsträme und Potenziale unter Berücksichtigung von Neu-, Aus- und Rückbau sowie der Instandsetzung*. 95 (2015).

93. Gassner, A., Lederer, J. & Fellner, J. Material stock development of the transport sector in the city of Vienna. *Journal of Industrial Ecology* **24**, 1364–1378 (2020).

94. Brinckerhoff, P. Chapter 4: Track Structure Design. in *Track Design Handbook for Light Rail Transit* vol. Second Edition (The National Academies Press, 2012).

95. Hanson, C. S., Noland, R. B. & Porter, C. D. Greenhouse gas emissions associated with materials used in commuter rail lines. *International Journal of Sustainable Transportation* **10**, 475–484 (2016).

96. Profillidis, V. A. *Railway Management and Engineering*. (Ashgate Publishing Limited, 2014).

97. Rose, J. G. *Introduction to Railway Infrastructure*. (2014).

98. Colorado Asphalt Pavement Association. *A guideline for the design and construction of asphalt parking lots in Colorado*. 16 (2016).

99. City of Missoula. *Part 4: Asphalt pavement design guide for the City of Miioula Missoula Parks and Recreation*. (2017).

100.

County of Los Angeles. *Chapter 22.112: Parking*. (2021).

101.

Glick, S., Shuler, S. & Guggemos, A. A. Life Cycle Analysis for Sustainable Development: A Case Study of Parking Lot Pavements. *International Journal of Construction Education and Research* **9**, 226–236 (2013).

102.

Minnesota Asphalt Pavement Association. *Asphalt Paving Design Guide*. 75 (2014).
